# Photokinetics of Bimolecular Reactions: Predictive Modelling of the Basic Bimolecular Photoreactions XXYY′(Φ,k) and XX′YY′(Φ,k)

**DOI:** 10.3390/molecules31173086

**Published:** 2026-09-02

**Authors:** Mounir Maafi

**Affiliations:** Leicester School of Pharmacy, De Montfort University, The Gateway, Leicester LE1 9BH, UK; mmaafi@dmu.ac.uk

**Keywords:** bimolecular photoreaction, photokinetics, Φ-order kinetics, quantum yield, predictive modelling, kinetic elucidation

## Abstract

The photokinetic behaviour of bimolecular photoreactions is usually considered to obey one of the three (zeroth, first, or second) orders of thermal kinetics. The underlying hypothesis here (*de facto* equating the kinetics of thermal and photoreactions) is not consistent with the major differences in the two physical systems (e.g., light and heat absorption by molecules are differently quantified). Also, while the rate laws of thermal reactions are, in principle, all analytically solvable, the non-linear ones of photoreactions are all (but one) unsolvable by known mathematical methods. This mathematical issue represents an important reason behind the underdevelopment of photokinetics along the general principles established in kinetics. To move beyond the current *status quo*, there is a need for a new strategy. A successful approach, previously confirmed for unimolecular photoreactions, is employed here, for the first time, to bimolecular photoreactions involving one or two reactants (X and $X^{\prime }$), one or two products ($Y^{\prime }$ and Y) and two (photo- and thermal) processes. It consists of proposing a general integrated rate law (or model explicit equation), testing and validating it by both excellent fitting of the *full kinetic traces* of the reaction species obtained by Runge–Kutta (RK) numerical integration and a linear relationship between theoretical, RK- and fitting-calculated values of the initial reaction rates. The features of the reactions in the testing set (more than 200 cases) spanned a wide range of possible reaction conditions and properties. The kinetic behaviour of bimolecular photoreactions was proven to be well described by a model equation involving the typical Φ-order kinetics, $\alpha \;Log\left(1+\beta e^{-\gamma t}\right)$, term. Furthermore, a detailed kinetic elucidation method was developed for the determination of the full set of reaction parameters for the two bimolecular photoreactions studied, $XXYY^{\prime }(\Phi ,k)$ and $XX^{\prime }YY^{\prime }(\Phi ,k)$. The model equation, the trends observed in reactivity with the various reaction parameters, and the elucidation method represent the first complete characterisation of the bimolecular photoreactions’ photokinetics ever published in the photochemistry literature. The present work contributes to the standardisation of photokinetics, its concepts and its investigative tools.

## 1. Introduction

Kinetics is an analytical tool for reaction dynamics that is widely applied in scientific, academic and engineering contexts. It can be used to target either the determination of the reaction parameters’ values and/or the unravelling of the mechanism undergone by the reaction, that is the pattern of the chemical transformations sustained by the involved molecular species. Kinetics is also employed to model and optimise the reaction behaviour to either predict the evolution of a reaction under given conditions or identify the optimal conditions for a specific target application (e.g., to maximise the reaction yield). Kinetics can also serve the unravelling of the reaction pathway through a mechanistic elucidation, usually based on measuring the effect of extrinsic variables (e.g., initial concentration) on the reaction dynamics. Kinetics is fundamentally based on a mathematical description of the physical system. The DNA of a reaction is held within a system of differential equations (the rate laws). The solutions of the rate laws (the integrated rate laws) give the formulations of the observables, i.e., the formulae expressing the variation with the reaction time (t) of the species concentrations ($C\left(t\right)$). These equations also hold the reaction rate constant (k) as an intrinsic parameter. In thermal kinetics, three basic integrated rate laws are defined as follows: the zeroth-order (as a linear function, $C\left(t\right)=C\left(0\right)-k\;t$), the first-order (mapped out by an exponential formula, $C\left(t\right)=C\left(0\right)e^{-k\;t}$), and the second-order kinetics (given by a reciprocal expression, $C\left(t\right)=C\left(0\right)/\left(1+C\left(0\right)k\;t\right)$).

The kinetics of photoreactions are most often described, in the literature, by the same integrated rate laws employed for thermal kinetics (i.e., defining zeroth-, first-, or second-order kinetics). The ubiquity of this approach does not, however, conceal or justify its inconsistency for the purpose, since both the rate laws and the integrated rate laws of thermal reactions are deprived of two essential parameters of light-driven reactions, the radiation intensity and the amount of the light absorbed, both directly influencing the overall rates of photoreactions (this aspect is further discussed in Section 2.2). In this context, it has already been established [1] that unimolecular photoreactions (phototransformation of one reactant into photoproducts, X→Y…) are faithfully described by Φ-order kinetics, a log-exp model equation expressing the concentration of the photoreactive species as a logarithmic formula bearing an exponential function in its argument, having a typical general formulation, $\alpha \;Log(1+\beta e^{-kt})$.

Better control and quantification of bimolecular photoreactions (involving two reactants, $X+X^{\prime }\to Y\ldots$) is still awaiting the development of holistic methods that integrate the latter two parameters (light absorption/intensity) into the rate laws of such photoprocesses (see Section 2.3), with perhaps the exception being a recent study [2] that identified the set of bimolecular photoreactions whose rate laws can analytically be solved. The closed-form integration of these rate laws established that the dynamics of that set of reactions irrevocably had a Φ-order kinetics character.

The relevance of photokinetics to modern technology has been evidenced in fields such as active biological molecules (for fine chemistry, medicine and pharmacy) [3,4,5,6,7,8,9,10], photocatalysis [11,12,13], greener and sustainable chemistry via organic photochemistry and its industrial applications [9,12,14,15,16,17,18,19,20,21,22], environmental research [23,24], continuous-flow photochemistry [25,26,27,28,29], photodimerisation [30,31,32], photoswitches [33,34,35], photopolymers and 3-D printing [36,37,38], and photosensitisation [39].

It is also worth noting that, while light-triggered bimolecular photoreactions have a long history (starting around 1870 [40,41,42,43,44,45]), procure environmental benefits and are cost effective, the efficacy of photochemically induced transformations is rather limited to lab experiments with very few direct applications in scaled-up processes [27,45,46]. Some of the likely reasons for such a situation in the pharmaceutical industry were attributed to a lack of equipment, regulatory experience, and photochemical expertise within the company/organisation [27].

Consistent standard formalisms for the kinetic analysis of bimolecular photoreactions and their homologous photosystems are also lacking throughout the literature. Their development will offer avenues to remove some of the hurdles faced by some technological processes. The present paper addresses this gap in the knowledge by proposing tested and validated tools and a consistent framework.

## 2. Results and Discussion

### 2.1. The Considered Bimolecular Photoreactions

**Scheme 1:**
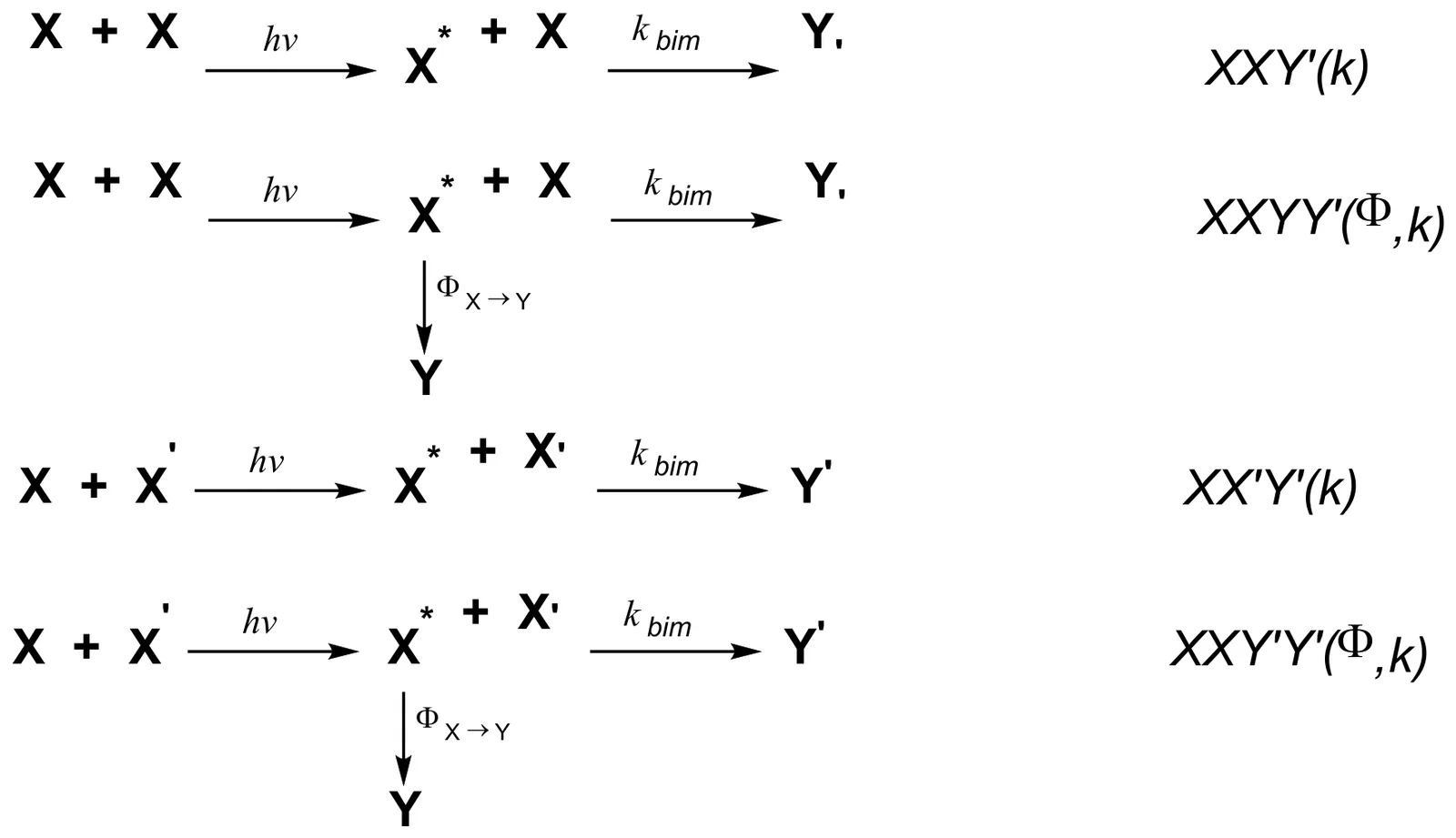
Basic bimolecular photoreactions.

Bimolecular reactions imply the presence of two reactants (X and $X^{\prime }$). In photochemistry, one of the reactants (e.g., X) is in an excited state ($X^{*}$) after the absorption of a photon (hv), and the other one (e.g., $X^{\prime }$) is in its ground state. The interaction between the two reactants yields one or several products (e.g., $Y^{\prime }$). The excited state, $X^{*}$, might well undergo alone a photoconversion to a different product (e.g., Y), indicating the occurrence of a unimolecular photoreaction of X (concomitant to the bimolecular photoreaction). When a single reactant is present ($X^{\prime }=X$), the aforementioned processes can similarly arise. The most ubiquitous basic reactions that we shall consider are depicted in Scheme 1.

To distinguish among the different reactions, a new labelling is proposed for the bimolecular photoreactions. It includes the reactants (XX or $XX^{\prime }$), the products ($Y^{\prime }$ or $YY^{\prime }$) and the thermal (k) and photochemical (Φ) processes involved, in addition to the light (hv) inducing the photoreaction. Accordingly, we have two straightforward reactions, $XXY^{\prime }(k)$ and $XX^{\prime }Y^{\prime }(k)$, proceeding by a light excitation of one reactant, followed by a thermal reaction of the two reactants. The second type includes two branched reactions, $XXYY^{\prime }(\Phi ,k)$ and $XX^{\prime }YY^{\prime }(\Phi ,k)$, where the excited reactant decays by two pathways (one purely photochemical and the other a thermal combination of $X^{*}$ with the second reactant).

The reaction between $X^{*}$ and $X^{\prime }$ is indicated, in Scheme 1, by the rate constant of the bimolecular photoreaction, $k_{bim}$. Whereas the direct photoconversion of X into Y is implied by the photochemical quantum yield, $\Phi_{X\to Y}^{\lambda_{irr}}$, which necessarily refers to a monochromatic excitation wavelength, $\lambda_{irr}$, absorbed by that species and which implicitly encompasses the losses along the X→Y pathway (e.g., emission ($X^{*}\to X+hv^{\prime }$) and non-radiative internal conversion and/or intersystem crossing, leading back to the initial molecule ($X^{*}\to X$)).

The theoretical rate laws of the bimolecular photoreactions shown in Scheme 1 must all be mathematically different. However, the literature variably accommodates these differences, as discussed in the next section.

### 2.2. Most Ubiquitous Kinetic Treatments in the Literature

The following two methods have generally been adopted to frame the kinetic behaviour of photoreactions: the steady state approach and the dynamic reaction method.

The former is not considered here as it imposes a hypothetical equilibrium between the produced and depleted molecules in the excited state (resulting in a net-zero rate of those species). This approach has been found to be useful for proposing relatively simple formulae for the *overall* quantum yield of the studied reaction. Even though the number of parameters involved in such equations is relatively low, some of them still need to be approximated or ignored altogether [47]. The required number of assumptions for the implementation of this approach means that concerns may be raised around the reliability of the obtained overall quantum yield values.

The kinetic method considers the reaction dynamics. It starts from the rate law of the reaction at hand, which reflects the continuous variation in the species concentrations over time. From this point of view, this method seems more consistent.

The most common approach to studying bimolecular photoreaction dynamics relies on the usage of either first- or second-order kinetic equations, established for thermal reactions [8,48,49,50,51,52,53,54,55,56,57,58,59,60,61]. The first-order equation is obtained from the second-order rate law if a much higher concentration of the second reactant ($X^{\prime }$ compared to that of X) is considered. Here, $C_{X^{\prime }}\left(t\right)$ is set to be constant throughout the reaction time, and, hence, the rate will be dependent on $C_{X}\left(t\right)$ only. In this case, the overall reaction is said to obey first-order kinetics (called pseudo-first-order kinetics). Examples of reciprocal second-order thermal kinetic models (i.e., the reaction rate is proportional to the square of the concentration) were also applied [50,55,56,62,63,64,65] for reactions involving a single reactant ($X=X^{\prime }$) or when the same initial concentration value is imposed on both reactants (i.e., $C_{X}\left(0\right)=C_{X^{\prime }}\left(0\right)$).

The handy equations above provide an easy and straightforward implementation of the kinetic data treatments, but, unfortunately, they do not deliver a coherent account of the dynamics of the considered bimolecular photoreactions. Many reasons underlie such a conclusion. The obvious arguments are the absence from the rate laws of thermal reaction kinetics of the radiation intensity, the amount of absorbed light and any distinction of the nature of the excitation light (mono- or polychromatic). A more serious reason relates to the mathematical formulation of the second-order rate law of thermal reactions. In the latter formulae, the molecules of X are effectively all potentially reactive with $X^{\prime }$ $(dC_{X}\left(t\right)/dt=-k_{∆}C_{X}\left(t\right)C_{X^{\prime }}\left(t\right)$), while in bimolecular photoreactions, only a fraction of the X molecules that reach an excited state are susceptible to reacting with $X^{\prime }$. This analysis shows, therefore, that the kinetic models describing thermal reactions are not suitable for the acutely different physical nature of bimolecular photoreactions—a conclusion reached and supported by many researchers [24,47,52,66,67,68].

In other approaches, the incident radiation intensity was explicitly included in the rate law of bimolecular photoreactions [55,56,58,59,60,61,69], but the difficulty in solving these rate laws meant that the temporal dependence of the reactive species were usually overlooked, focusing the treatments of the data on the early stages of the kinetic traces [61,70].

One option to reduce the complexity of the standard non-linear rate laws (which involve radiation intensity and absorption terms) consisted in expanding the factor, $10^{-\varepsilon \;l\;C}$, by power series expansion, though usually considering only its first-order form, to finally obtain the approximated, εlC, term and, hence, linearising the rate law [8]. Often the application of this approach ignores the condition imposed on using the first-order power series expansion, that is, the requirement of small values of the coefficient in the exponent of the power number [1] (i.e., εlC≤0.01)—a condition representing a serious experimental limitation.

The variety of the kinetic approaches and/or the mathematical formulations of the rate laws do indicate a lack of consensus in the community, an absence of identified kinetic orders of the reactions, and a lack of explicit formulae for the bimolecular photoreactions’ integrated rate laws.

In fact, the few bimolecular photoreactions’ rate laws that can be analytically solved have been identified in a recent paper [2]. These rate laws include coefficients for the radiation intensity and the absorbed light fraction. Only the rates of two bimolecular photoreactions ($XX^{\prime }Y^{\prime }(k)$ and $XX^{\prime }YY^{\prime }(\Phi ,k)$) are solvable by closed-form integration under the following four conditions: the excitation is carried out by a strictly monochromatic and collimated light beam, only the photoreactive reactant X absorbs the irradiation light, the initial concentration of $X^{\prime }$ is high, and the concentration of X belongs to the linearity range of its absorbance calibration graph. The Φ-order kinetics was established for these reactions’ behaviours. Also, it was proven that the rate constants of their overall bimolecular photoreactions were different from the rate constants of the thermal bimolecular processes.

Otherwise, the non-linear rate laws of bimolecular photoreactions are, in general, not possibly integrable by any known conventional mathematical method. As such, they follow the pattern identified for unimolecular photoreactions [1].

### 2.3. The Theoretical Rate Law of Bimolecular Photoreactions

The bimolecular photoprocesses considered in the present study are based on the data collected spectrophotometrically, i.e., on a time scale of a second and longer. In this sense, the sub-second processes are not observed and, therefore, the rate constants of the bimolecular photoreactions of Scheme 1 are time independent. The general rate equation of such a bimolecular photoreaction (Scheme 1) takes into account the light absorption by the molecules present in the reactive medium at time t [1], and the thermal reaction between a reactant in its excited state ($X^{*}$) and the second ground state reactant ($X^{\prime }$). The rate of the reaction is, hence, proportional to the fraction ($P_{a_{X}}^{\lambda_{irr}}$) of the incident light absorbed by the photoactive X to produce $X^{*}$ [2], the rate constant of the bimolecular photoreaction ($k_{bim}$, expressed in $M^{-1}$, whose unit is different from that of pure thermal bimolecular reaction), the absorptivities of the species (ε, $mol^{-1}\;dm^{3}\;cm^{-1}$), and the time-variable concentrations (e.g., $C_{X^{\prime }}^{\Delta \lambda ,T}\left(t\right)$) [2]. The rate may also encompass the concomitant unimolecular photoreaction of the excited state species ($X^{*}$) leading to the formation of Y, if this photoprocess is active. Finally, the rate includes the sum of terms over the considered range of wavelengths of the lamp ($\Delta \lambda =\lambda_{b}-\lambda_{a}$, in nm) when the reaction is exposed to a polychromatic irradiation beam. The global rate law ($r_{X}^{\Delta \lambda ,T}\left(t\right)$ expressed in $M\;s^{-1}$) for the timely variation in reactant X’s concentration ($C_{X}^{\Delta \lambda ,T}\left(t\right)$, in M) can be laid out for $XX^{\prime }YY^{\prime }(\Phi ,k)$ (Equation (1)) for a slab-shaped reactor, where the vigorously-stirred reactive medium is maintained at a constant temperature (T) and constantly subjected to a collimated light beam.

(1) $$r_{X}^{\Delta \lambda ,T}\left(t\right)=\frac{dC_{X}^{\Delta \lambda ,T}\left(t\right)}{dt}=-\int_{\lambda_{a}}^{\lambda_{b}}\left(k_{bim}C_{X^{\prime }}^{\Delta \lambda ,T}\left(t\right)+\Phi_{X\to Y}^{\lambda_{irr}}\right)P_{a_{X}}^{\lambda_{irr}}\left(t\right)d\lambda$$

Equation (1) equally applies if the light is monochromatic, hence, removing from Equation (1) the integral over λ and replacing Δλ with $\lambda_{irr}$. If the unimolecular photoreaction is absent, $\Phi_{X\to Y}^{\lambda_{irr}}$ is assigned a zero value. When the unimolecular is to be studied alone (where $X^{\prime }$ is not added to the reactive medium), then $C_{X^{\prime }}^{\Delta \lambda ,T}\left(t\right)=k_{bim}=0$ in Equation (1). For the latter case, the resulting Equation (1) is equivalent to that of the primary photoprocess’ rate [1,71,72,73,74]. When the bimolecular photoreaction involves a single type of reactant, $X^{\prime }=X$, Equation (1) is only a function of $C_{X}^{\Delta \lambda ,T}\left(t\right)=C_{X^{\prime }}^{\Delta \lambda ,T}\left(t\right)$.

The integro-differential rate equations describing the dynamics of the remaining species of the reactions can also be laid out (in the case of reaction $XXYY^{\prime }(\Phi ,k)$, Equation (2) is equivalent to Equation (1)).

(2) $$r_{X^{\prime }}^{\Delta \lambda ,T}\left(t\right)=\frac{dC_{X^{\prime }}^{\Delta \lambda ,T}\left(t\right)}{dt}=-k_{bim}C_{X^{\prime }}^{\Delta \lambda ,T}\left(t\right)\int_{\lambda_{a}}^{\lambda_{b}}P_{a_{X}}^{\lambda_{irr}}\left(t\right)d\lambda$$

(3) $$r_{Y}^{\Delta \lambda ,T}\left(t\right)=\frac{dC_{Y}^{\Delta \lambda ,T}\left(t\right)}{dt}=\int_{\lambda_{a}}^{\lambda_{b}}\Phi_{X\to Y}^{\lambda_{irr}}P_{a_{X}}^{\lambda_{irr}}\left(t\right)d\lambda$$

(4) $$r_{Y^{\prime }}^{\Delta \lambda ,T}\left(t\right)=\frac{dC_{Y^{\prime }}^{\Delta \lambda ,T}\left(t\right)}{dt}=k_{bim}C_{X^{\prime }}^{\Delta \lambda ,T}\left(t\right)\int_{\lambda_{a}}^{\lambda_{b}}P_{a_{X}}^{\lambda_{irr}}\left(t\right)d\lambda =-r_{X^{\prime }}^{\Delta \lambda ,T}\left(t\right)$$

The fraction of the incident light absorbed by X ($P_{a_{X}}^{\lambda_{irr}}\left(t\right)$, Equation (5)) is a non-linear function of the absorbances of both X and the medium ($A_{X}^{\lambda_{irr},T}\left(t\right)$ and $A_{tot}^{\lambda_{irr}T}\left(t\right)$, respectively). $P_{a_{X}}^{\lambda_{irr}}\left(t\right)$ (Equation (5)) has the same unit as the incident radiation intensity, $P_{0}^{\lambda_{irr}}$ ($einstein\;dm^{-3}\;s^{-1}$). Therefore, the dimension analysis of the right-hand side of Equations (1)–(4) is equal to that of $r^{\Delta \lambda ,T}\left(t\right)$, since an einstein is a mole of photons and the quantum yield is dimensionless.

(5) $$P_{a_{X}}^{\lambda_{irr}}\left(t\right)=\frac{A_{X}^{\lambda_{irr},T}\left(t\right)}{A_{tot}^{\lambda_{irr},T}\left(t\right)}P_{0}^{\lambda_{irr}}\left(1-10^{-A_{tot}^{\lambda_{irr},T}\left(t\right)}\right)=P_{0}^{\lambda_{irr}}A_{X}^{\lambda_{irr},T}\left(t\right)\frac{1-10^{-A_{tot}^{\lambda_{irr},T}\left(t\right)}}{A_{tot}^{\lambda_{irr},T}\left(t\right)}$$

The total absorbance ($A_{tot}^{\lambda_{irr}}\left(t\right)$ in Equation (5)), measured for the reactive medium at $\lambda_{irr}$, is expressed by a sum of the species absorbances.

(6) $$A_{tot}^{\lambda_{irr},T}\left(t\right)=A_{X}^{\lambda_{irr},T}\left(t\right)+A_{Y}^{\lambda_{irr},T}\left(t\right)+A_{X^{\prime }}^{\lambda_{irr},T}\left(t\right)+A_{Y^{\prime }}^{\lambda_{irr},T}\left(t\right)$$

The dimensionless absorbance, $A_{j}^{\lambda_{irr},T}\left(t\right)=\varepsilon_{j}^{\lambda_{irr}}l_{irr}C_{j}^{\lambda_{irr},T}\left(t\right)$, measured at time t (in s) and $\lambda_{irr}$, is the product of the optical path length of the excitation light inside the reactive medium, ($l_{irr}$, in cm), the decadic molar absorption coefficients, ε, and the concentration of the species at time t, $C_{j}^{\lambda_{irr},T}\left(t\right)$ (in $M\;or\;mol\;dm^{-3}$). Owing to the fact that irradiation by the light source and observation (or monitoring) by the spectrophotometer might be performed across two different optical path lengths ($l_{irr}$ and $l_{obs}$), the values of the absorbances $A^{\lambda_{irr},T}\left(t\right)$ (occurring in Equation (5)) can be evaluated by the counterpart values of $A^{\lambda_{obs}}\left(t\right)$ recorded by the spectrophotometer, using the conversion formula $A^{\lambda_{irr}}\left(t\right)=A^{\lambda_{obs}}\left(t\right)l_{irr}/l_{obs}$. The mandatory condition applied to all of the absorbances is that they must fall within the linearity ranges of their specific calibration graphs. This condition implies that the Beer–Lambert law must apply [1], otherwise Equation (5) is invalid and so would be Equations (1)–(4).

The main issue facing the photokinetics of bimolecular photoreactions stems essentially from the simple fact that their integro-differential systems of equations characterising such photoreactions cannot be analytically solved. Furthermore, they have not been solved in the literature by any adequate numerical method treating integro-differential equations. Thus far, a single study [75] solved the integro-differential rate of a unimolecular photoreaction by high-order positivity-preserving approximation schemes, but only for the linear first-order form of the rate (dC(t)/dt=−∑k C(t)), i.e., not considering the non-linear forms of Equations (1)–(4). The impossibility of a closed-form derivation of the integrated rate law also arises for bimolecular photoreactions driven by monochromatic light.

In the present work, Equations (1)–(4) are numerically integrated, notably by replacing the integrals with a sum over the considered range of λ at one-nanometre increments (for polychromatic irradiation). This technique has proven very efficient for both irradiation options (mono- and polychromatic light) [1,76]. The simulated kinetic data of the bimolecular photoreactions were obtained on a homemade programme, coding for the fourth-order Runge–Kutta (RK) numerical integration, written using the Excel VBA platform. The choice of RK is justified in Section 2.7.

It is also useful to define the equations of the metric (the initial reaction rate), where the theoretical ones are determined from Equations (1)–(4) when t=0. Accordingly, the theoretical initial rate of reactant X takes the following expression that yields negative values,

(7) $$Theo:r_{X}^{\Delta \lambda ,T}\left(0\right)={Theo:r}_{0,X}^{\Delta \lambda ,T}=-\sum_{\lambda_{irr}=\lambda_{a}}^{\lambda_{b}}\left(k_{bim}C_{X^{\prime }}^{\Delta \lambda ,T}\left(0\right)+\Phi_{X\to Y}^{\lambda_{irr}}\right)P_{a_{X}}^{\lambda_{irr}}\left(0\right)$$

which is equivalent to the sum of the positive initial rates of the Y and $Y^{\prime }$ reactions, but these are multiplied by a negative sign.

(8) $${Theo:r}_{0,X}^{\Delta \lambda ,T}={Theo:r}_{0,X^{\prime }}^{\Delta \lambda ,T}-{Theo:r}_{0,Y}^{\Delta \lambda ,T}=-{Theo:r}_{0,Y}^{\Delta \lambda ,T}-{Theo:r}_{0,Y^{\prime }}^{\Delta \lambda ,T}$$

### 2.4. Evaluation of the Classical Kinetic Treatments Applied to the Data of Bimolecular Photoreactions

**Figure 1:**
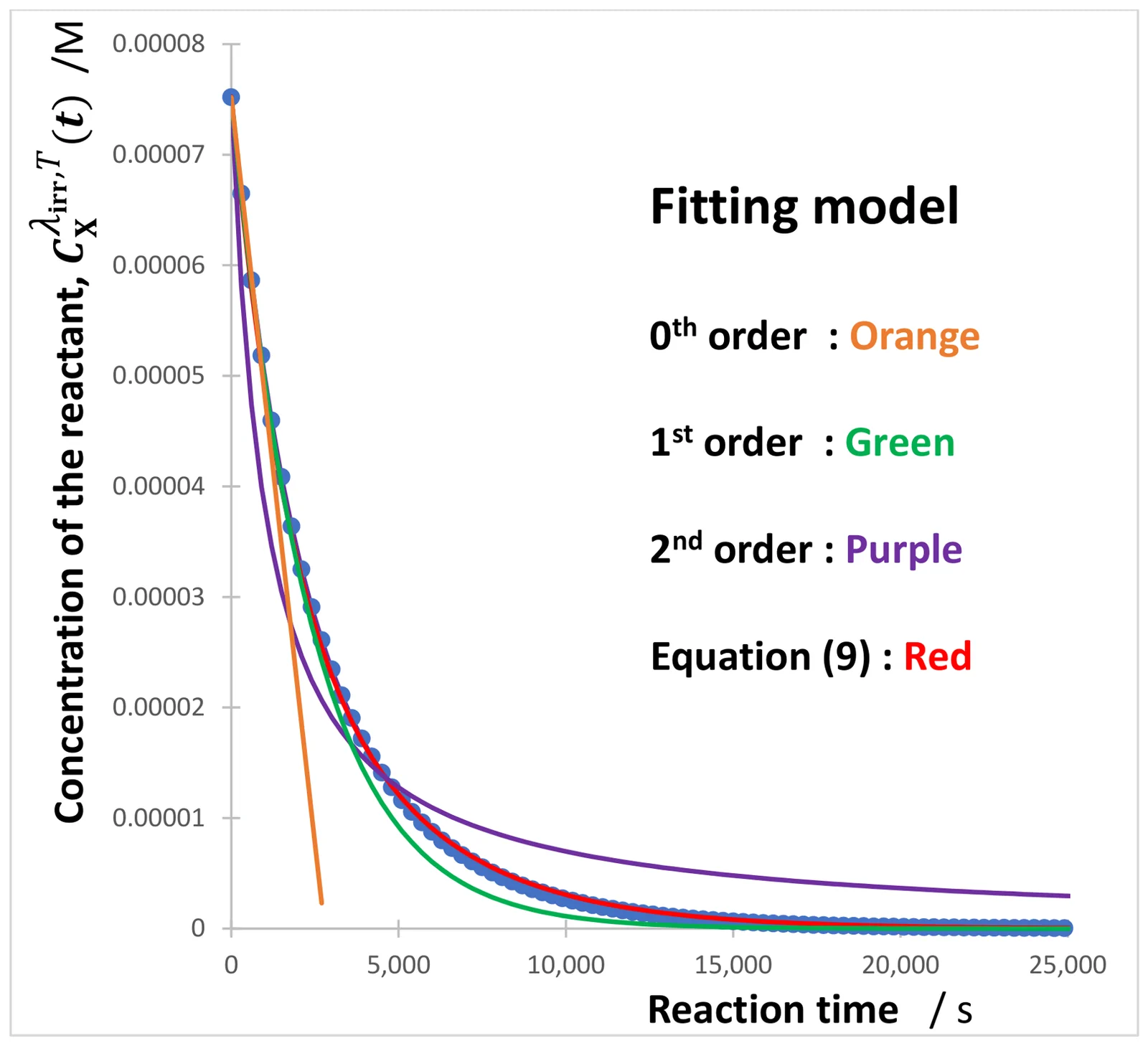
An attempt to fit the kinetic data of the photoactive reactant of a bimolecular XX′Y′(k) photoreaction subjected to a monochromatic light beam by the classical thermal reaction models and Equation (9). The kinetic data (dots) were generated by a Runge–Kutta numerical integration for reactions characterised by the following extrinsic parameters: $C_{X}^{\lambda_{irr},T}\left(0\right)=7.52\times 10^{-5}\;M,$ $C_{X^{\prime }}^{\lambda_{irr},T}\left(0\right)=1.1\times 10^{-4}\;M,$ $P_{0}^{\lambda_{irr}}=1.7\times 10^{-3}\;einstein\;dm^{-3}\;s^{-1},$ and $l_{irr}=0.36\;cm$; intrinsic parameters: $k_{bim}=0.34\;M^{-1},$ $\varepsilon_{X}^{\lambda_{irr}}=20,953\;M^{-1}\;cm^{-1},$ and $\varepsilon_{X^{\prime }}^{\lambda_{irr}}=1770\;M^{-1}\;cm^{-1}$. The fitting parameters indicating the goodness of fit of Equation (8) are $r^{2}=0.9999$. $SSE=7.29\times 10^{-14}$ and $RMSE=1.58\times 10^{-8}$.

The treatment of the kinetic data of bimolecular photoreactions is usually performed using thermal kinetic models. Even when overlooking their lack of physicochemical consistency, such models still do not seem to necessarily be applicable to all cases. For illustration, the data of the simplest $XX^{\prime }Y^{\prime }(k)$ shown in Figure 1 are not interpreted by any of the equations belonging to the most common thermal kinetic reaction orders when the whole kinetic trace is considered. It could be argued that a given equation might well partially apply to the set of data making up the trace (e.g., the first stages of the reaction, Figure 1), but this justification would implicitly disqualify that equation because it con-firms that the equation cannot represent the whole reaction kinetics (i.e., cannot be applied to the whole trace). Similar inconsistent fitting with first- and second-order models was evidenced for the photocatalytic decolourisation of rhodamine B by tungsten trioxide in aqueous solutions [52].

Owing to this discrepancy, some authors have suggested that bimolecular photoreactions might obey fractional orders [52,75,77,78]. The latter approach does not ascribe a unique fractional order to these reactions and, more importantly, does not fundamentally justify why bimolecular photoreactions would kinetically behave according to the proposed fractional order (i.e., the fractional order does not follow from the standard rate laws of bimolecular photoreactions, e.g., Equation (1)). The approach seems to be mainly looking for possible equations that can fit the kinetic data, while considering the bimolecular photoreactions to behave as a pure thermal process (since for a fractional first-order kinetic model it was stipulated [52] that the rate law is similar to the first-order kinetic equation, except that the derivative of the concentration with respect to the reaction time is not an integer but a real number). In a sense, whether integer or fractional orders are envisaged, these approaches suffer from the same limitation, that is, they do not truly describe the physical photosystems at hand (since the light absorption is ignored in the rate laws).

As stipulated in Section 2.2, to the best of our knowledge, bimolecular photoreactions should rather obey Φ-order kinetics. Φ-Order kinetics tends to behave as a quasi-linear (zeroth-order kinetics) or an exponential pattern (first-order kinetics) at limit values of the absorbance (respectively, at a very high or a very low absorbance values of the photoreactive species, X) [1,2]. Incidentally, when analytically integrable, the rate laws of unimolecular and bimolecular photoreactions, as set in Equations (1)–(4), did not mathematically yield the reciprocal formula characterising the second-order kinetics [1,2].

Conceptually, Equations (1)–(4) describe the general case. In doing so, they do not, in principle, satisfy all of the conditions stated for the cases where analytical integration is possible (Section 2.2), and so Equations (1)–(4) remain unsolvable by conventional mathematical methods. As a consequence, the specific kinetic order of the corresponding bimolecular photoreactions cannot be identified with accuracy and, thus far, it remains unknown. This leads us to a general statement, that is, the conventional route for developing a framework of kinetic investigation, starting from solving the rate law of the reaction studied, is not affordable to the kinetics of most unimolecular and bimolecular photoreactions (only very few cases break away from this rule). Other strategies must be envisaged to allow for investigation of these dynamic photosystems according to the same general standards, rules and concepts adopted in classical thermal kinetics.

### 2.5. Methodology for the Elaboration of a Valid Model Equation for the Integrated Rate Law

A full analytical solution for the pure thermal $XX^{\prime }Y^{\prime }(k)$ and $XXY^{\prime }(k)$ bimolecular reactions (not initiated by light), involving linear differential equations, has fully been achieved [79]. However, the analytical integration of Equation (1), characterising bimolecular photoreactions, is not possible. In this context, it is worth acknowledging a general method that has previously been proposed to build consistency, as well as allowing for quantification of unimolecular photoreactions in an accurate manner [1,72,80,81]. The strength of this method is embedded within the five criteria it should satisfy: The method consists of testing and validating (a) proposed explicit integrated rate laws for the photoreaction system studied. For that purpose, (b) the proposed explicit formulae must apply to a wide variety of reaction cases (usually more than 100 that differ by their specific reaction conditions and their species’ features). (c) The explicit formulae must imperatively show an excellent fit of the “*whole kinetic traces*” of the species involved in each reaction case (stating from t=0 up to the end of the reaction at t=∞). In addition, (d) the initial rate of the reactant ($r_{0,X}^{∆\lambda {\;or\;\lambda }_{irr},T}$) derived theoretically ($Theo:r_{0,X}$) must be equal or very close (<10% difference) to those either calculated from the proposed explicit equation (e.g., $Fit:r_{0,X}$ obtained by differentiation of the explicit equation) or generated by the numerical integration (e.g., $RK:r_{0,X}$). If the explicit model formula is not always valid for the set of traces considered, then (e) it is imperative to clearly define its limits of application (generally by defining its validity over a specific domain of total absorbance values [1]). Finally, the ultimate confirmation of the usefulness and validity of the proposed integrated rate law is provided by (f) the development of a kinetic elucidation method, where each intrinsic and unknown reaction feature is identified by a unique equation that can be calculated using extrinsic features (that are experimentally accessible) and/or previously calculated intrinsic features by this elucidation procedure. The confirmation of the validity of the elucidation method must be carried out by testing it on RK-traces, where during the application of the elucidation method, the intrinsic/inaccessible parameters are considered unknown. The calculated intrinsic/unknown parameters’ values obtained by the kinetic elucidation method should be close to their counterparts’ originally used to feed the RK-calculation. Hence, if a proposed explicit formula satisfies the five aforementioned criteria ((b)–(f)), it can be considered a validated model formula useful for application to the kinetic data of the photoreaction considered. Practically, such an explicit (surrogate) model formula (such as Equation (9)) plays the same role as an analytically derived integrated rate law (since it conforms to and reproduces the highly accurate RK data). From this standpoint, it does not need to be tested on traces with experimental errors as much as the analytically derived equations (e.g., the monoexponential of the first-order kinetics) are not tested on such data to prove their validity.

The present methodology circumvents the possibility of a lack of generality of a proposed formula, as well as possible discrepancies generated by using a reduced set of experimental traces and data sets to test a proposed formula (as usually observed in the photokinetic literature where a model formula is deemed valid if it fits the data of one or a few experiments).

### 2.6. The Proposed Integrated Rate Equations

It is obvious that the explicit formula to be proposed for the bimolecular photoreaction (criterion (a)) must have the Φ-order character embedded within its formulation. This follows from the fact that the only analytically integrated rate laws thus far known for unimolecular and bimolecular photoreactions are described by Φ-order kinetics [1,2,71]. The Φ-order is specific to photoreactions. It is mathematically formulated as a logarithmic function bearing an exponential in its argument, typically standing as $\alpha \;Log\left(1+\beta \;e^{-\;\gamma \;t}\right)$, where α, β and γ are constants.

For the general case of bimolecular photoreactions, laid out in Scheme 1, the following integrated rate law (the explicit model equation) is proposed for reactant X:

(9) $$C_{X}^{\Delta \lambda ,T}\left(t\right)=\left(u_{1}\;-u_{2}\;\right)\;u_{3}\;\frac{Log\left(1+u_{4}\;e^{-\;u_{5}\;t}\right)+Log\left(1+u_{6}\;e^{-\;u_{7}\;t}\right)}{u_{1}-u_{2}\;u_{8}\;Log\left(1+u_{9}\;e^{-\;u_{5}\;t}\right)}\;\;$$

The $u_{1}$–$u_{9}$ coefficients are constants. The variation in the reactant’s concentration starts from $C_{X}^{\lambda_{irr},T}\left(0\right)$ (at t=0) and tends to a zero value after a long reaction time (t=∞). Equation (9) is suitable for all four types of bimolecular photoreactions—$XXY^{\prime }(k)$*,* $XX^{\prime }Y^{\prime }(k)$*,* $XXYY^{\prime }(\Phi ,k)$, and $XX^{\prime }YY^{\prime }(\Phi ,k)$ (Scheme 1)—when exposed to either mono- or polychromatic light. Equation (9) reduces to a single Φ-order term when $u_{6}=u_{9}=0$ and to a ratio of mono-Φ-order terms when $u_{4}=0$. The former case ($u_{6}=u_{9}=0$) allows for the incorporation of the special cases of reactions $XX^{\prime }Y^{\prime }(k)$ and $XX^{\prime }YY^{\prime }(\Phi ,k)$, when considered under the conditions stated in Section 2.2 and for which the rates were integrated in a closed form, into this general equation (Equation (9)) [2]. The formulation of the explicit equation when $u_{4}=0$ is analogous to that describing the kinetics of the thermal bimolecular reaction when $C_{X}^{\lambda_{irr},T}\left(0\right)\neq C_{X^{\prime }}^{\lambda_{irr},T}\left(0\right)$, which involves a ratio of monoexponential functions [1].

The explicit model equation relative to the concentration of the product generated by either reaction $XX^{\prime }Y^{\prime }(k)$ or $XXY^{\prime }(k)$ is given by the following:

(10) $$C_{Y^{\prime }}^{\Delta \lambda ,T}\left(t\right)=C_{X}^{\Delta \lambda ,T}\left(0\right)-C_{X}^{\Delta \lambda ,T}\left(t\right)\;$$

The formulae for the products of reaction $XX^{\prime }YY^{\prime }(\Phi ,k)$ are less straightforward because Y and $Y^{\prime }$ are produced by different concurrent processes.

For the unimolecular photoconversion of X into Y (i.e., in the absence of $X^{\prime }$), the classical mono-Φ-order equation is sufficient for monochromatic light irradiation [1,2,71,72] when only the reactant absorbs (Equation (11)), but for the case where the reactive X is not the only absorbing species in the medium, Equation (14) applies.

(11) $$C_{Y}^{\lambda_{irr},T}\left(t\right)=\frac{1}{\varepsilon_{X}^{\lambda_{irr}}\;l_{irr}}\;Log\left(1+\left(10^{\varepsilon_{X}^{\lambda_{irr}}\;l_{irr}\;C_{X}^{\lambda_{irr},T}\left(0\right)}-1\right)\;e^{-k_{X\to Y}\;t}\right)\;$$

The concentration formula for $Y^{\prime }$ in both the $XX^{\prime }Y^{\prime }\left(k\right)$ and $XX^{\prime }YY^{\prime }\left(\Phi ,k\right)$ reactions, takes the following form:

(12) $$C_{Y^{\prime }}^{\Delta \lambda ,T}\left(t\right)=C_{X^{\prime }}^{\Delta \lambda ,T}\left(0\right)-C_{X^{\prime }}^{\Delta \lambda ,T}\left(t\right)$$

The concentration of the photochemically inert reactant, $X^{\prime }$, has the following formula:

(13) $$C_{X^{\prime }}^{\Delta \lambda ,T}\left(t\right)=C_{X^{\prime }}^{\Delta \lambda ,T}\left(0\right)-C_{Y^{\prime }}^{\Delta \lambda ,T}\left(t\right)=C_{X^{\prime }}^{\Delta \lambda ,T}\left(0\right)-\left(C_{X}^{\Delta \lambda ,T}\left(0\right)-C_{X}^{\Delta \lambda ,T}\left(t\right)\right)+C_{Y}^{\Delta \lambda ,T}\left(t\right)$$

The dynamic evolution of the concentration of product Y in the $XX^{\prime }YY^{\prime }\left(\Phi ,k\right)$ reactions, ($C_{Y}^{\Delta \lambda ,T}\left(t\right)=0$ for reactions $XX^{\prime }Y^{\prime }\left(k\right)$), is mapped out with Equation (14) irrespective on how many species absorb the incident light at time t [1,75,76]. It is here formulated by two mono-Φ-order terms for generality (as it is unlikely that this equation encompasses more than two terms, but it could be reduced to a single term, i.e., $\omega_{4}=0$).

(14) $$C_{Y}^{\Delta \lambda ,T}\left(t\right)=\omega_{0}+\omega_{1}\;Log\left(1+\omega_{2}\;e^{-\omega_{3}\;t}\right)+\omega_{4}\;Log\left(1+\omega_{5}\;e^{-\omega_{6}\;t}\right)$$

Accordingly, at the end of the reaction ($t\to \infty ,\;C_{X}^{\Delta \lambda ,T}\left(\infty \right)=0$), the following identity must be verified:

(15) $$C_{Y}^{\Delta \lambda ,T}\left(\infty \right)+C_{Y^{\prime }}^{\Delta \lambda ,T}\left(\infty \right)=C_{X}^{\Delta \lambda ,T}\left(0\right)$$

When $C_{X}^{\Delta \lambda ,T}\left(0\right)=C_{X^{\prime }}^{\Delta \lambda ,T}\left(0\right),$ the final concentrations of $X^{\prime }$ and Y in $XX^{\prime }YY^{\prime }\left(\Phi ,k\right)$ or $XXYY^{\prime }\left(\Phi ,k\right)$ reactions are equal, $C_{X^{\prime }}^{\Delta \lambda ,T}\left(\infty \right)=C_{Y}^{\Delta \lambda ,T}\left(\infty \right)$ (consider Equation (13) at t→∞).

In addition to the concentration formulae, it is important to define the initial rate equation for the reactant. The latter is derived for X by differentiation of Equation (9) at time t=0, as follows:

(16) $$Fit:r_{0,X}^{\Delta \lambda ,T}=-\;\frac{\left(u_{1}\;-u_{2}\;\right)\;u_{3}}{ln(10)}\left(\frac{\frac{u_{5}\;u_{4}}{1+u_{4}}+\frac{u_{7}\;u_{6}}{1+u_{6}}\;}{u_{1}-u_{2}\;u_{8}\;Log\left(1+u_{9}\right)}\;+\right.\;\;\;\;\;\;\;\;\;\;\;\left.\frac{\left(Log\left(1+u_{4}\right)+Log\left(1+u_{6}\right)\right)\;u_{2}\;u_{8}\left(\frac{u_{9}\;u_{5}}{1+u_{9}}\right)}{{\left(u_{1}-u_{2}\;u_{8}\;Log\left(1+u_{9}\right)\right)}^{2}}\right)\;$$

The initial rates of the remaining species are similarly obtained by differentiations of the corresponding Equations (10)–(13) and which satisfy Equation (8).

### 2.7. The Set of Testing Data

The most effective low-cost and resource- and time-saving method of collecting a high number of kinetic traces for significantly different reaction cases, as required by criterion (b) (in Section 2.5), is to simulate the reaction behaviour using a numerical integration method (NIM).

The choice of a NIM is crucial for the success of the whole method. An adequate NIM must be capable of delivering data that have lower errors than the experimental data, and, hence, it is comparable with a theoretical formula. It turns out that the fourth-order Runge–Kutta method (RK) is one of the best NIMs for such a purpose. It has been previously adopted [1] and will also be employed in this paper. In addition to the fact that RK provides the $C\left(t\right)$ traces, while the rate law is not integrable, its errors are well below the experimental ones, and it reproduces with high accuracy the traces generated from the analytically derived integrated rate laws (e.g., the exponential curves of the first-order kinetics and the Φ-order traces of the bimolecular photoreactions [1,2]). Hence, the RK-generated traces are as good as those produced by the analytically derived integrated rate laws. Therefore, practically, this means that the RK-traces can serve to test the explicit model formulae, as a test on integrated rate laws or on pure reaction data would do (with virtually few or no experimental errors).

We are, therefore, able to work on a relatively large number of sample data for many different reactions—a process that is hardly achieved by experiment, especially since RK generates the $C\left(t\right)$ traces of all of the species present, whether appearing or disappearing, in the reactive medium, creating a set of data that might not always be easily accessible experimentally.

Finally, the RK simulation provides both the $C\left(t\right)$ and $A\left(t\right)$ traces for the individual species. It also avoids possible discrepancies in the generalisation of the proposed equation applicability that might be linked to using a reduced set of experimental data to test that formula (as is usually performed in photokinetics practice).

The RK kinetic data that are generated from the true and physically consistent rate laws represent an excellent tool for predictive modelling of bimolecular photoprocesses.

One point needs to be kept in mind when using RK calculations in the present context, that is, the data are generated assuming that the absorbances of the species present in the reactor have values falling within the corresponding linearity ranges of their respective calibration graphs (this is inherent to NIM calculations in this context). In practice, this condition is generally satisfied if $A_{tot}^{\lambda_{irr},T}\left(t\right)\leq 0.5$ [1], which is a conservative value when an experimental determination of the linearity ranges is not feasible. The drawback of performing the (RK) numerical method is the impossibility of knowing the formula and/or value of the rate constant of the photoprocess studied.

### 2.8. The Validation of the Model Equation

**Figure 2:**
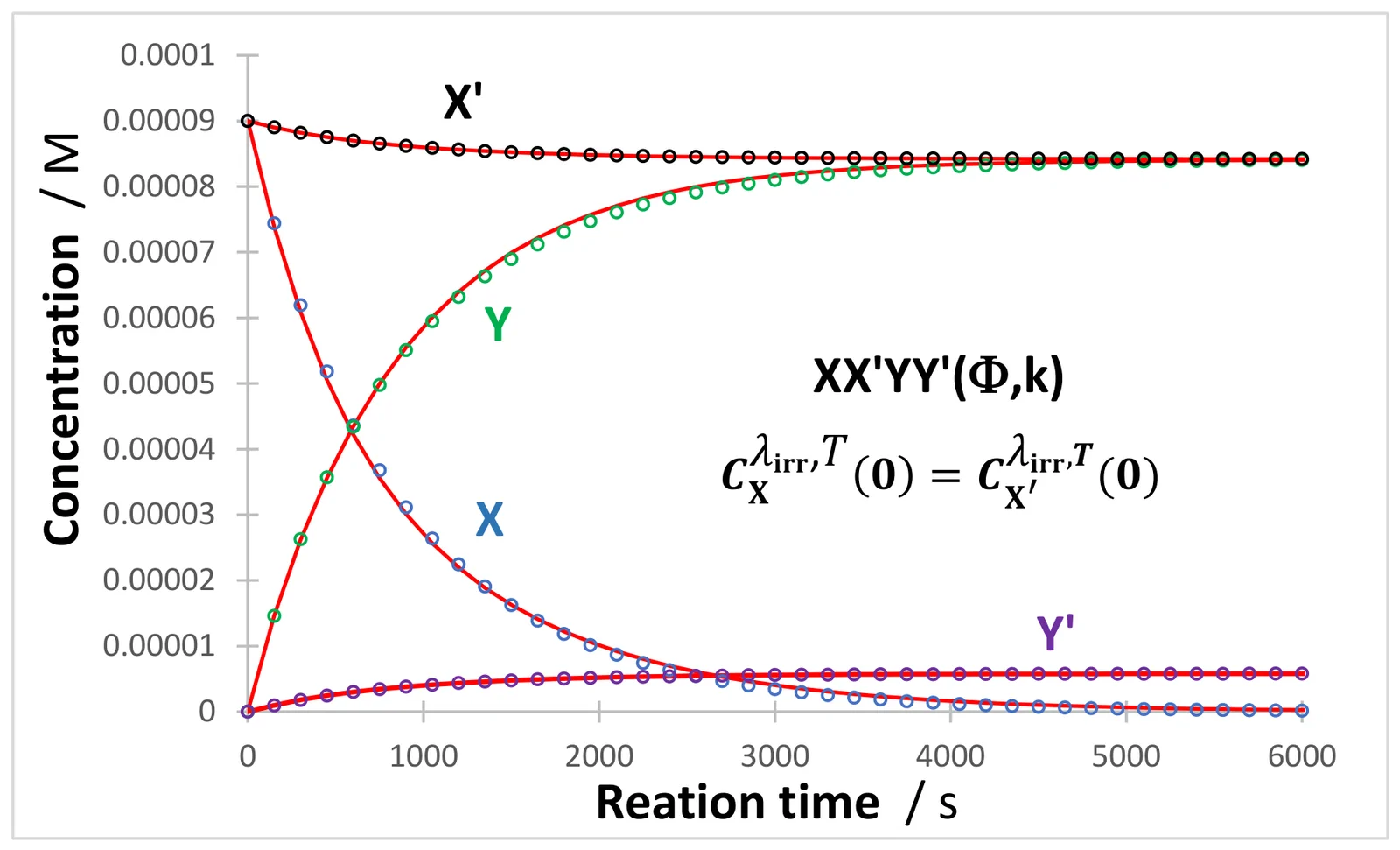
Fitting of the RK-traces (circles) belonging to the four species of an $XX^{\prime }YY\prime (\Phi ,k)$ (in the present case, this is equivalent to $XXYY^{\prime }(\Phi ,k))$ reaction under monochromatic irradiation. The reaction’s extrinsic parameters are $C_{X}^{\lambda_{irr},T}\left(0\right)=9\times 10^{-5}M=C_{X^{\prime }}^{\lambda_{irr},T}\left(0\right)$, $P_{0}^{\lambda_{irr}}=1.6\times 10^{-4}\;einstein\;dm^{-3}\;s^{-1}$, and $l_{irr}=0.39\;cm$, and its intrinsic parameters are $k_{bim}=2.83\;M^{-1},$ $\Phi_{X\to Y}^{\lambda_{irr}}=0.00416,$ $\varepsilon_{X}^{\lambda_{irr}}=2586\;M^{-1}\;cm^{-1},$ $\varepsilon_{X^{\prime }}^{\lambda_{irr}}=3587\;M^{-1}\;cm^{-1},$ $\varepsilon_{Y}^{\lambda_{irr}}=10,211\;M^{-1}\;cm^{-1},$ and $\varepsilon_{Y^{\prime }}^{\lambda_{irr}}=7421\;M^{-1}\;cm^{-1}$. The fitting curves (red lines) correspond to the following series of equations used: Equation (10) (X), Equation (13) ($X^{\prime }$), Equation (14) (Y in the reaction), and Equation (12) ($Y^{\prime }$).

The explicit model equations have been tested on 200 different reactions (criterion (b) in Section 2.5). The RK-generated traces belong to reactions obeying one of the four types of mechanisms depicted in Scheme 1. The photoreactive systems contrasted by different combinations of intrinsic ($10^{3}\leq \varepsilon \leq 45\times 10^{3}$, $0.01\leq k_{bim}\leq 5$, and $0\leq \Phi_{X\to Y}\leq 1$) and extrinsic ($10^{-6}\leq C_{X}\left(0\right)\leq 10^{-3}$, $10^{-6}\leq C_{X^{\prime }}\left(0\right)\leq 10^{-3}$, $10^{-7}\leq P_{0}\leq 10^{-3}$, and $0.1\leq l_{irr}\leq 3$) parameters characterising each reaction case. The reliability of Equation (9) is indicated by an excellent fit of all of the RK-simulated concentration traces considered (criterion (c), Section 2.5). The fitting with the Levenberg–Marquardt algorithm is smooth and quick despite both the relatively high number ($u_{1}-u_{9}$) of fitting parameters, the non-linear fitting process, and fitting the whole curved traces. Several features assessed the goodness of fit, including the linear correlation between the RK data and those produced by the fitting equation (with r>0.99 and intercepts practically equal to zero) and the low values of both the SSE (sum of squared errors $<10^{-10}$) and the RMSE (root mean square error $<10^{-6}$). An illustration of the species’ traces of the bimolecular photoreactions is shown in Figure 2. The initial fitting values were randomly selected until a good fit was obtained. The numerical integration step size was varied depending on the relative speed of the reaction (ranging between 5 s for fast processes and 3000 s for slow ones). However, the values of the tolerance for function, the termination tolerance parameter, and the tolerance on constraint violation were kept the same for all fittings ($10^{-15}$).

The above results testify to the validity of the explicit equations to consistently describe the kinetic behaviour of the bimolecular photoreactions listed in Scheme 1. It is also demonstrated that the kinetics of these reactions clearly show a *Φ*-order character. This represents the first proof of its kind in the field of photokinetics. The explicit Equations (9)–(14) are also set as the first general equations ever reported in the literature for such reactions.

To test further the validity of the explicit equations, it is necessary to compare the initial reaction rate values (criterion (d) in Section 2.5).

The $r_{0}$ serves equally as a quantification metric in kinetics. Usually, the reaction rate constant is the chosen metric in thermal kinetics. However, the concept of the rate constant is dual for bimolecular photoreactions as the rate constant of the overall photoreaction, $k_{r}$, has a different value of that corresponding to the rate constant $k_{bim}$. From the previously established [2] analytically derived equations, $k_{bim}$ is only one among several factors making up the equation for $k_{r}$. Nonetheless, since Equation (1) is not integrable, the explicit equation for $k_{r}$ remains unknown for bimolecular photoreactions. Also, there is an impossibility to decide on the absolute value of $k_{r}$ from Equation (9). Therefore, $r_{0}$ emerges as the sole accessible kinetic metric available from the analysis of the traces, allowing for the assessment of reactivity ($k_{bim}$ is determined from $r_{0}$, as we shall see in the next section). It is important to underline that the absolute value of ${Fit:r}_{0,X}$ (Equation (16)) is not affected by the identifiability issue impinging on the values of the $u_{1}–u_{9}$ parameters. Indeed, the values of this set of fitting parameters depends on the choice of the initial values selected for this set of parameters at the start of the non-linear fitting process. In other words, different initial values will yield a different set of values for the $u_{1}$–$u_{9}$ parameters, but with the resulting Equation (9) fitting well the RK-trace. The identifiability problem is recurrent in the kinetic analysis for the explicit equations (such as Equation (9)), as well as for the analytically derived integrated rate laws, if the number of parameters is relatively high [1,82]. The good fit of the kinetic data by the considered equation becomes a necessary but not a sufficient condition [1]. Hence, in all cases, a kinetic elucidation method is mandatory to fully solve the identifiability problem and the kinetics (as stipulated in point (f) of Section 2.5). However, this unidentifiability of the values of the $u_{1}$–$u_{9}$ parameters does not affect/modify the value of ${Fit:r}_{0,X}$. This is an additional reason why $r_{0}$ arose as the metric for explicit (non-analytically derived) equations [1,72,79,80,81].

**Figure 3:**
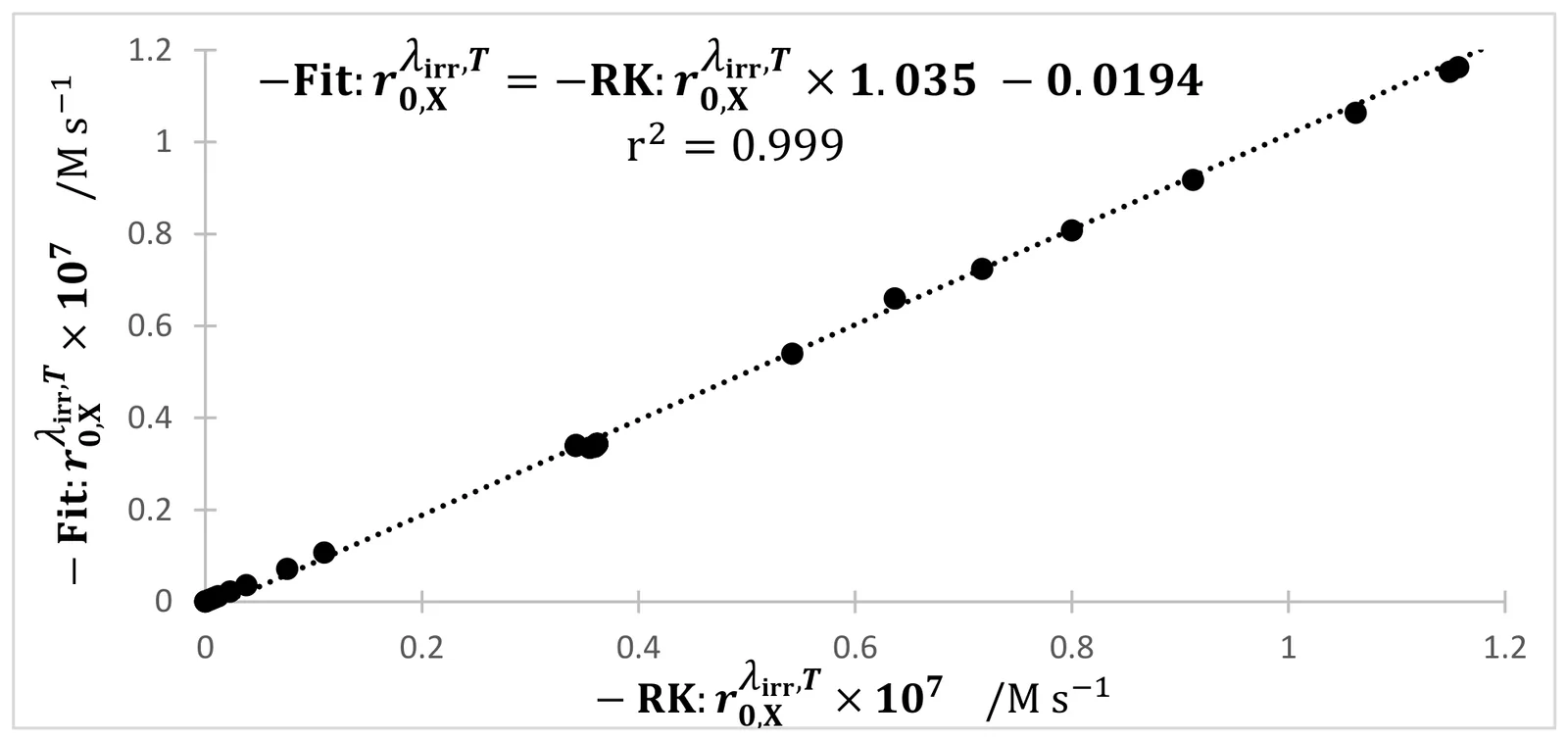
An excellent linear correlation characterises the plot of the initial reaction rates of the four species obtained theoretically, calculated from the fitting equations, and determined from the RK calculation.

The plot of ${Fit:r}_{0}$ against ${RK:r}_{0}$ and ${Theo:r}_{0}$ (Figure 3) yields a linear relationship ($r^{2}≅1\;and\;intercept≅0$), confirming both that the equations used for the fitting, Equations (9), (10), (12) and (13), are useful for the description of the photokinetics of bi- molecular reactions (Scheme 1) and that the initial rates calculated from the fitting traces for the four species are reliable investigation metrics.

It is relevant to mention that Equations (9), (10) and (12)–(14) are applicable to the fitting of the species’ traces for all reaction cases (i.e., have no detected limits for their application) irrespective of the specific species’ properties and/or reaction conditions (criterion (e) in Section 2.5), provided that the concentrations used correspond to the absorbances’ linearity ranges of the species (e.g., absorbance does not exceed a value of 0.5 at any reaction time and any wavelength [1], as stated above).

Here, we discuss another limitation of Equation (9), that is, for the $u_{1}$–$u_{9}$ parameters it may be suggested that the lack of $u_{1}$–$u_{9}$ identities could jeopardise the whole kinetic analysis process. This assumption would be true if the elucidation of the kinetics is not achievable. In fact, despite the exact formulae of the $u_{1}$–$u_{9}$ parameters not being known, the equations set out in Section 2.6 are still very useful for a full kinetic analysis, i.e., achieving the purpose of a kinetic investigation. A proof is provided in the next section.

### 2.9. The Kinetic Elucidation Method

The purpose of the kinetic elucidation method is to fully solve the kinetics by determining the whole set of values of the reaction features. The features’ values must be unique, i.e., each feature is obtained in a unique way and identified by a specific equation (criterion (f) in Section 2.5). A full elucidation is only possible when the irradiation is both monochromatic (at $\lambda_{irr}$) and collimated [1]. Also, the individual trace of the photoactive reactant (X) must be known (a full elucidation is not possibly achievable from the total absorbance trace only, or when the light is polychromatic).

For the case of our reactions (Scheme 1), they might be characterised by up to 10 parameters. Four of those are extrinsic parameters, i.e., directly accessible by the experiment. These are the initial concentrations of the reactants $C_{X}^{\lambda_{irr},T}\left(0\right)$ and $C_{X^{\prime }}^{\lambda_{irr},T}\left(0\right)$, the optical path length crossed by the monochromatic/collimated excitation light inside the reactor, $l_{irr}$, and the incident radiation intensity, $P_{0}^{\lambda_{irr}}$, which is determined by actinometry (a set of kinactinometers covering 220–580 nm, where previously proposed [1]). The are up to six intrinsic parameters, as follows: the four absorption coefficients at $\lambda_{irr}$ ($\varepsilon_{X}^{\lambda_{irr}}$, $\varepsilon_{X^{\prime }}^{\lambda_{irr}}$, $\varepsilon_{Y}^{\lambda_{irr}}$, and $\varepsilon_{Y^{\prime }}^{\lambda_{irr}}$), the rate constant of the bimolecular photoreaction, $k_{bim}$, and the photochemical quantum yield, $\Phi_{X\to Y}^{\lambda_{irr}}$.

Because the extrinsic parameters are known, the elucidation will only be concerned by the determination of the intrinsic parameters.

The absorptivities of X and $X^{\prime }$ are obtained from the linearity ranges of their respective individual calibration graphs that would be performed in the same solvent and temperature in which the bimolecular photoreaction will later be realised. The linearity ranges must be defined to serve the selection of the possible initial concentrations (of both reactants) that will be used for the bimolecular photoreaction.

The absorption coefficient $\varepsilon_{Y}^{\lambda_{irr}}$ can be determined from the final absorbance recorded after the total depletion of X in an irradiated solution of X alone (without $X^{\prime }$ being present in the reactive medium, i.e., the unimolecular photoreaction X→Y). The final absorbance in this case, $A_{tot}^{\lambda_{irr}}\left(\infty \right)=A_{Y}^{\lambda_{irr}}(\infty )=\varepsilon_{Y}^{\lambda_{irr}}l_{irr}C_{X}^{\lambda_{irr},T}\left(0\right)$, can be used to plot the calibration graph of Y (by varying the value of $C_{X}^{\lambda_{irr},T}\left(0\right)$) and the determination of $\varepsilon_{Y}^{\lambda_{irr}}$.

The value of $\Phi_{X\to Y}^{\lambda_{irr}}$ is determined from the fitting parameter ($k_{X\to Y}$) of Equation (11) when fitting the trace of the previous unimolecular photoreaction (X→Y), in the case where only X absorbs ($\varepsilon_{Y}^{\lambda_{irr}}=0$). Indeed, the explicit expression of $k_{X\to Y}$ has been established analytically [1,71], and when rearranged yields the following:

(17) $$\Phi_{X\to Y}^{\lambda_{irr}}=\frac{1}{k_{X\to Y}}\varepsilon_{X}^{\lambda_{irr}}P_{0}^{\lambda_{irr}}l_{irr}\ln\left(10\right)$$

In the case that both the reactant and product of the unimolecular X→Y absorb the incident light, the concentration trace of X is fitted to a previous explicit equation developed for this reaction [73,74] and the quantum yield is similarly extracted from the formula of the rate constant. Alternatively, Equation (14) with a single mono-Φ-order term can be used to fit the photoactive reactant trace of any unimolecular photoreaction (X→Y), $C_{X}^{\Delta \lambda ,T}\left(t\right)=\omega_{0}+\omega_{1}Log\left(1+\omega_{2}e^{-\omega_{3}t}\right)$, as established for unimolecular reactions under irradiation by monochromatic light [72]. In this case, the quantum yield is determined from the value of the initial reaction rate obtained from the fitting combined with its theoretical expression, as follows:

(18) $$\Phi_{X\to Y}^{\lambda_{irr}}=-\frac{{Fit:r}_{0,X}^{\lambda_{irr},T}}{P_{a_{X}}^{\lambda_{irr}}\left(0\right)}$$

Now, we will deal with the kinetic data of the bimolecular $XX^{\prime }YY^{\prime }(\Phi ,k)$ photoreaction. The rate constant of the thermal bimolecular reaction is extracted from the formula of the theoretical initial rate of X. According to a rearranged Equation (7), $k_{bim}$ should be equal to the following:

(19) $$k_{bim}=\frac{-1}{C_{X^{\prime }}^{\Delta \lambda ,T}\left(0\right)}\left(\frac{{Theo:r}_{0,X}^{\lambda_{irr},T}}{P_{a_{X}}^{\lambda_{irr}}\left(0\right)}+\Phi_{X\to Y}^{\lambda_{irr}}\right)$$

By replacing $P_{a_{X}}^{\lambda_{irr}}\left(0\right)$ with its expression (Equation (5)), ${Theo:r}_{0,X}^{\lambda_{irr},T}$ takes its equivalent value, i.e., the value of ${Theo:r}_{0,X}^{\lambda_{irr},T}$ is equal to ${Fit:r}_{0,X}^{\lambda_{irr},T}$, which is obtained from the fitting of Equation (9) to the concentration trace of reactant X when the bimolecular photoreaction is performed under monochromatic irradiation. Using the already known quantities, we deduce the formula of $k_{bim}$ as being only defined by known coefficients, as follows:

(20) $$k_{bim}=\frac{-1}{C_{X^{\prime }}^{\Delta \lambda ,T}\left(0\right)}\left(\Phi_{X\to Y}^{\lambda_{irr}}+\frac{{Fit:r}_{0,X}^{\lambda_{irr},T}\left(\varepsilon_{X}^{\lambda_{irr}}C_{X}^{\lambda_{irr},T}\left(0\right)+\varepsilon_{X^{\prime }}^{\lambda_{irr}}C_{X^{\prime }}^{\lambda_{irr},T}\left(0\right)\right)}{\varepsilon_{X}^{\lambda_{irr}}C_{X}^{\lambda_{irr},T}\left(0\right)P_{0}^{\lambda_{irr}}\left(1-10^{-\varepsilon_{X}^{\lambda_{irr}}C_{X}^{\lambda_{irr},T}\left(0\right)-\varepsilon_{X^{\prime }}^{\lambda_{irr}}C_{X^{\prime }}^{\lambda_{irr},T}\left(0\right)}\right)}\right)$$

The knowledge of $\Phi_{X\to Y}^{\lambda_{irr}}$ and $k_{bim}$ allows for the calculation of the initial rates of the $XX^{\prime }YY^{\prime }(\Phi ,k)$ bimolecular photoreaction products, as deduced from Equations (3) and (4), without necessarily accessing the actual concentration kinetic traces of these species.

(21) $$r_{Y}^{\lambda_{irr},T}\left(0\right)=\Phi_{X\to Y}^{\lambda_{irr}}P_{a_{X}}^{\lambda_{irr}}\left(0\right)$$

(22) $$r_{Y^{\prime }}^{\lambda_{irr},T}\left(0\right)=k_{bim}C_{X^{\prime }}^{\lambda_{irr},T}\left(0\right)P_{a_{X}}^{\lambda_{irr}}\left(0\right)$$

Similarly, the value of $r_{X^{\prime }}^{\lambda_{irr},T}\left(0\right)$ can be determined from either Equation (1) or (8). It is interesting to observe that because the final concentrations of the products are fractions of $C_{X}^{\lambda_{irr},T}\left(0\right)$, as stated in Equation (15), their explicit equations can be indicated by the following expressions:

(23) $$C_{Y}^{\lambda_{irr},T}\left(\infty \right)=-C_{X}^{\lambda_{irr},T}\left(0\right)\frac{r_{Y}^{\lambda_{irr},T}\left(0\right)}{r_{X}^{\lambda_{irr},T}\left(0\right)}=C_{X}^{\lambda_{irr},T}\left(0\right)\frac{\Phi_{X\to Y}^{\lambda_{irr}}}{\Phi_{X\to Y}^{\lambda_{irr}}+k_{bim}C_{X^{\prime }}^{\lambda_{irr},T}\left(0\right)}$$

(24) $$C_{Y^{\prime }}^{\lambda_{irr},T}\left(\infty \right)=-C_{X}^{\lambda_{irr},T}\left(0\right)\frac{r_{Y^{\prime }}^{\lambda_{irr},T}\left(0\right)}{r_{X}^{\lambda_{irr},T}\left(0\right)}=C_{X}^{\lambda_{irr},T}\left(0\right)\frac{k_{bim}C_{X^{\prime }}^{\lambda_{irr},T}\left(0\right)}{\Phi_{X\to Y}^{\lambda_{irr}}+k_{bim}C_{X^{\prime }}^{\lambda_{irr},T}\left(0\right)}$$

Consequently, we can calculate the value of the final concentration of $X^{\prime }$, as follows:

(25) $$C_{X^{\prime }}^{\lambda_{irr},T}\left(\infty \right)=C_{X^{\prime }}^{\lambda_{irr},T}\left(0\right)-C_{Y^{\prime }}^{\lambda_{irr},T}\left(\infty \right)$$

The last unknown to be determined by the kinetic elucidation method is the intrinsic absorptivity of $Y^{\prime }$ at $\lambda_{irr}$.

For the reaction $XX^{\prime }Y^{\prime }(k)$, the procedure is handy if we consider the traces belonging to the reaction where $C_{X}^{\lambda_{irr},T}\left(0\right)\leq C_{X^{\prime }}^{\lambda_{irr},T}\left(0\right)$ and assuming a complete depletion of X. Then, $\varepsilon_{Y^{\prime }}^{\lambda_{irr}}$ is calculated from a rearranged equation of the final total absorbance of the bimolecular photoreaction (measured at t=∞, Equation (6)), where $C_{Y^{\prime }}^{\lambda_{irr},T}\left(\infty \right)=C_{X}^{\lambda_{irr},T}\left(0\right)$, as follows:

(26) $$\varepsilon_{Y^{\prime }}^{\lambda_{irr}}=\frac{1}{C_{X}^{\lambda_{irr},T}\left(0\right)}\left(\frac{1}{l_{irr}}A_{tot}^{\lambda_{irr},T}\left(\infty \right)-\varepsilon_{X^{\prime }}^{\lambda_{irr}}\left(C_{X^{\prime }}^{\lambda_{irr},T}\left(0\right)-C_{X}^{\lambda_{irr},T}\left(0\right)\right)-\varepsilon_{Y^{\prime }}^{\lambda_{irr}}C_{X}^{\lambda_{irr},T}\left(0\right)\right)$$

Following the same procedure, the value of $\varepsilon_{Y}^{\lambda_{irr}}$ is obtained for the reaction XXY(k) from the following simple formula:

(27) $$\varepsilon_{Y}^{\lambda_{irr}}=\frac{A_{tot}^{\lambda_{irr},T}\left(\infty \right)}{C_{X}^{\lambda_{irr},T}\left(0\right)l_{irr}}$$

For the reaction $XX^{\prime }YY^{\prime }(\Phi ,k)$, the value of $\varepsilon_{Y^{\prime }}^{\lambda_{irr}}$ can be determined from the final total absorbance of this bimolecular photoreaction (measured at t=∞), because the final concentrations of $X^{\prime }$, Y, and $Y^{\prime }$, as well as the absorption coefficients of X, $X^{\prime }$ and Y at $\lambda_{irr}$, are all known (as, respectively, obtained from Equations (23)–(25) and the description provided at the beginning of this elucidation method). The calculation of the value of $\varepsilon_{Y^{\prime }}^{\lambda_{irr}}$ is, therefore, straightforward using Equation (6) (with $C_{X}^{\lambda_{irr},T}\left(\infty \right)=0$ and assuming that Y, $X^{\prime }$ and $Y^{\prime }$ all absorb at $\lambda_{irr}$).

(28) $$\varepsilon_{Y^{\prime }}^{\lambda_{irr}}=\frac{\frac{1}{l_{irr}}A_{tot}^{\lambda_{irr},T}\left(\infty \right)-\varepsilon_{X^{\prime }}^{\lambda_{irr}}C_{X^{\prime }}^{\lambda_{irr},T}\left(\infty \right)-\varepsilon_{Y}^{\lambda_{irr}}C_{Y}^{\lambda_{irr},T}\left(\infty \right)}{C_{Y^{\prime }}^{\lambda_{irr},T}\left(\infty \right)}$$

Accordingly, all extrinsic and, especially, intrinsic parameters can be uniquely defined by the overall procedure. Also, the present elucidation method proves efficient in solving the kinetics of the considered bimolecular photoreactions despite the lack of the explicit formulae of the Equation (9) parameters ($u_{1}$–$u_{9}$). The present elucidation method is, so far, unmatched in the literature on the photokinetics of bimolecular photoreactions.

As an example of its application, let us consider an $XX^{\prime }YY^{\prime }(\Phi ,k)$ reaction with the properties indicated in Table 1. The above elucidation method is doubly applied, firstly to the data obtained from the RK calculation to prove the validity of the method and, secondly, using the fitting results to demonstrate the method’s applicability in a realistic situation.

The results for both the RK and fitting data are characterised by relatively low errors compared with the theoretical values of the intrinsic parameters (Table 1), indicating high performance of the kinetic elucidation method.

### 2.10. The Effects of the Reactants’ Initial Concentrations and the Optical-Path Length Values on the Reactivity of the Bimolecular Photoreactions

The shape of the trace, for a given reaction, might change with an increase in the concentration of either the X or $X^{\prime }$ reactants. Such a possibility is formally offered by the three effective options in which Equation (9) can explicitly be formulated, as discussed in Section 2.6. The change in the trace shape is sustained by the previously reported properties of the Φ-order kinetics of the unimolecular photoreactions, where a high initial concentration of the reactant would yield a linear-type of trace, whereas at relatively low initial concentration values, the curve of the trace tends to fit an exponential profile. This typical behaviour has also been observed for bimolecular photoreactions whose integrated rate laws are analytically derived [2]. Even though not previously proven for the general case of bimolecular photoreactions, such changes in kinetic behaviour would conceivably be expected, as confirmed by the options supported by Equation (9). These changes in kinetic behaviour could be viewed as changes in the reaction order. As such, this information can palliate the lack of rationalisation relative to the change in reaction order (the so-called order-change puzzle) reported in the literature for bimolecular photoreactions [47,83,84] using the same unique set of four equations (Equations (9) and (12)–(14)) for the four species of the reactions (Scheme 1). The effect of increasing the initial concentration of either reactant can be quantified in the following two ways: by the initial reactants’ rates or by any features relative to the transformation of X after a given time.

According to Equations (1)–(4) of reaction $XXYY^{\prime }(\Phi ,k)$, a change in the initial concentration $C_{X}^{\Delta \lambda ,T}\left(0\right)$ is directly expressed by a change occurring to the amount of light absorbed, $P_{a_{X}}^{\lambda_{irr}}\left(t\right).$ This is true for the three (X, Y and $Y^{\prime }$) species of the reaction (as for reaction $XXYY^{\prime }(\Phi ,k)$, $X^{\prime }$ is absent and, hence, no Equation (2)). Therefore, at the initial time, $P_{a_{X}}^{\lambda_{irr}}\left(0\right)$ (according to Equation (5)) depends on the product $C_{X}^{\Delta \lambda ,T}\left(0\right)\times PKF(0)$, since both $A_{X}^{\Delta \lambda ,T}\left(0\right)$, $A_{tot}^{\lambda_{irr},T}\left(t\right)$ and $PKF\left(0\right)=\left(1-10^{-A_{tot}^{\lambda_{irr},T}\left(t\right)}\right)/A_{tot}^{\lambda_{irr},T}\left(t\right)$ depend on $C_{X}^{\Delta \lambda ,T}\left(0\right).$ The same trend is expected for reaction $XX^{\prime }YY^{\prime }(\Phi ,k)$ (with $C_{X^{\prime }}^{\Delta \lambda ,T}\left(0\right)$ kept constant).

A change in $C_{X^{\prime }}^{\Delta \lambda ,T}\left(0\right)$ for reaction $XX^{\prime }YY^{\prime }(\Phi ,k)$, with $C_{X}^{\Delta \lambda ,T}\left(0\right)$ remaining invariable, can be seen as inducing mathematically the same effect as described above, since both $C_{X}^{\lambda_{irr},T}\left(0\right)$ and $C_{X^{\prime }}^{\lambda_{irr},T}\left(0\right)$ multiply PKF(0) in Equations (1), (2) and (4). However, for product Y (Equation (3)), the evolution of $r_{0,Y}^{\Delta \lambda ,T}$ follows that of PKF(0) (not $C_{X^{\prime }}^{\lambda_{irr},T}\left(0\right)\times PKF(0)$).

It is, hence, important to review the variation in $P_{a_{X}}^{\lambda_{irr}}\left(0\right)$ with a change occurring in one concentration, either $C_{X}^{\lambda_{irr},T}\left(0\right)$ or $C_{X^{\prime }}^{\Delta \lambda ,T}\left(0\right)$, while the other one is kept constant.

**Figure 4:**
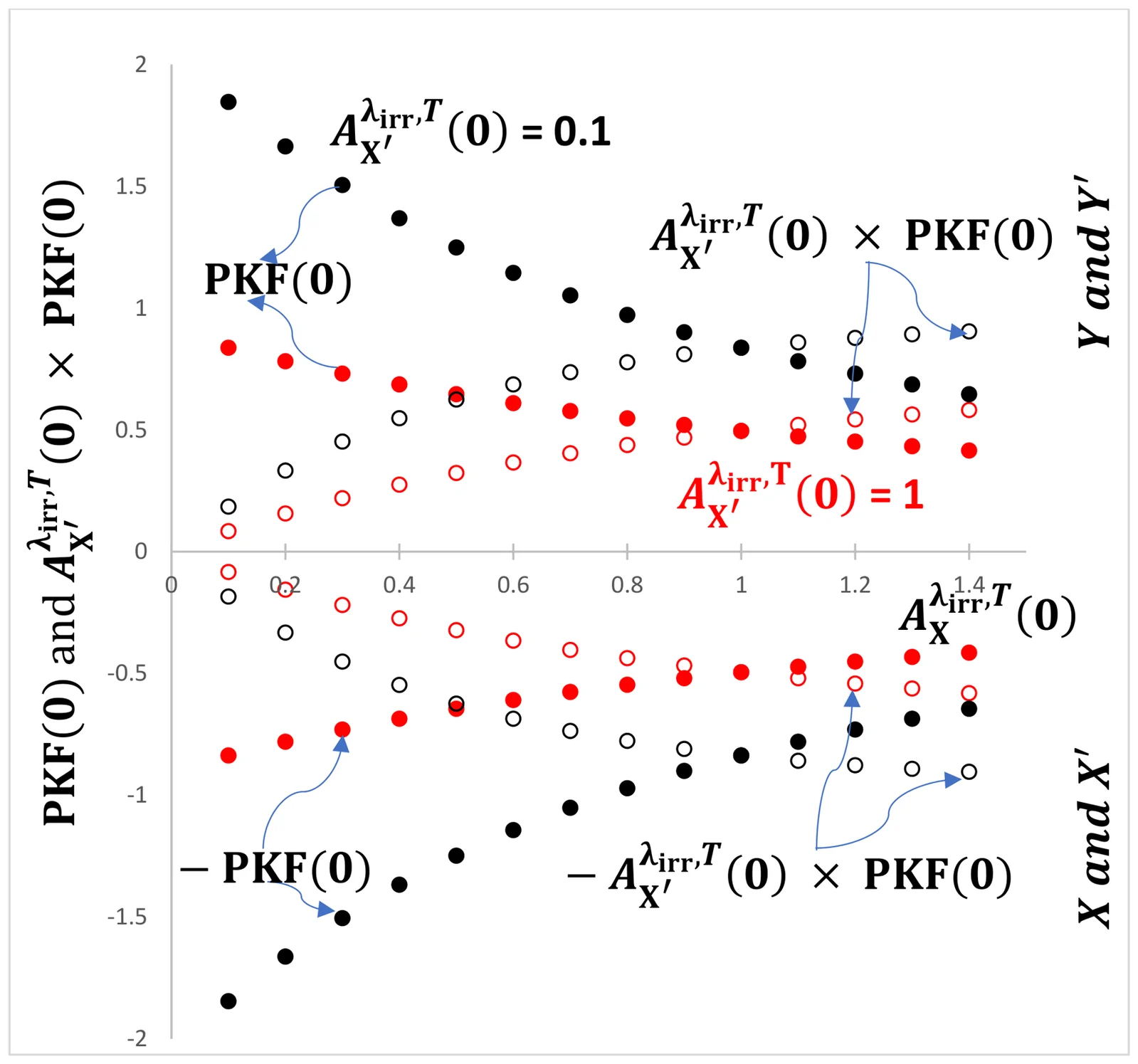
Evolutions of both the photokinetic factor, PKF(0), (dots), and the product, $PKF(0)\times A_{X^{\prime }}^{\lambda_{irr},T}\left(0\right)$, (circles), with increasing values of $A_{X}^{\lambda_{irr},T}\left(0\right)$ (i.e., increasing $C_{X}^{\lambda_{irr},T}\left(0\right)$). The variations in the above quantities are considered for two initial absorbances of $X^{\prime }$: the data for $A_{X^{\prime }}^{\lambda_{irr},T}\left(0\right)=0.1$ are presented in red, whereas those for $A_{X^{\prime }}^{\lambda_{irr},T}\left(0\right)=1$ appear in black. In the case of an $XX^{\prime }YY^{\prime }(\Phi ,k)$ reaction, the top section of the plot concerns the positive values of the above quantities of the products $Y\;and\;Y^{\prime }$, whereas the bottom section includes the negative values of their homologue quantities of the reactants $X\;and\;X^{\prime }$ (Equations (3)–(4) and (1)–(2), respectively).

The variations in PKF(0) and the product $PKF(0)\times A_{X^{\prime }}^{\Delta \lambda ,T}\left(0\right)$ with increases in the values of $A_{X^{\prime }}^{\Delta \lambda ,T}\left(0\right)$ are shown for the general case in Figure 4. As $A_{X^{\prime }}^{\Delta \lambda ,T}\left(0\right)$ is a function of $C_{X^{\prime }}^{\Delta \lambda ,T}\left(0\right)$, the variation in $PKF(0)\times A_{X^{\prime }}^{\Delta \lambda ,T}\left(0\right)$ has the same overall trend as that of $PKF(0)\times C_{X^{\prime }}^{\Delta \lambda ,T}\left(0\right)$, and that of $PKF(0)\times C_{X}^{\Delta \lambda ,T}\left(0\right)$ is similar to that of $PKF(0)\times A_{X}^{\Delta \lambda ,T}\left(0\right)$, with the latter case corresponding to $P_{a_{X}}^{\lambda_{irr}}\left(0\right)$.

Accordingly, the initial rate of reactant X ($r_{0,X}^{\Delta \lambda ,T}$) is expected to increase with an increase in $C_{X}^{\lambda_{irr},T}\left(0\right)$ (with $C_{X^{\prime }}^{\Delta \lambda ,T}\left(0\right)$ being constant) due to an increase in the value of $P_{a_{X}}^{\lambda_{irr}}\left(0\right)=PKF(0)\times A_{X}^{\Delta \lambda ,T}\left(0\right)$. However, the evolution of $r_{0,X}^{\Delta \lambda ,T}$ when $C_{X^{\prime }}^{\Delta \lambda ,T}\left(0\right)$ increases depends on the relative magnitude between $\Phi_{X\to Y}^{\lambda_{irr}}$ and the product $k_{bim}C_{X^{\prime }}^{\Delta \lambda ,T}\left(t\right).$ If their ratio ($k_{bim}C_{X^{\prime }}^{\Delta \lambda ,T}\left(t\right)/\Phi_{X\to Y}^{\lambda_{irr}}$) is too big, $r_{0,X}^{\Delta \lambda ,T}$ increases as does $PKF(0)\times A_{X^{\prime }}^{\Delta \lambda ,T}\left(0\right)$. However, for a very small ratio, $r_{0,X}^{\Delta \lambda ,T}$ will decrease, adopting the pattern of PKF(0). For Y, at the initial time, an increase in $C_{X^{\prime }}^{\Delta \lambda ,T}\left(0\right)$ (with a constant $C_{X}^{\Delta \lambda ,T}\left(0\right)$) causes a decrease in $P_{a_{X}}^{\lambda_{irr}}\left(0\right)$ (due to a decrease in PKF(0)) and, hence, a decrease in $r_{0,Y}^{\Delta \lambda ,T}.$

**Figure 5:**
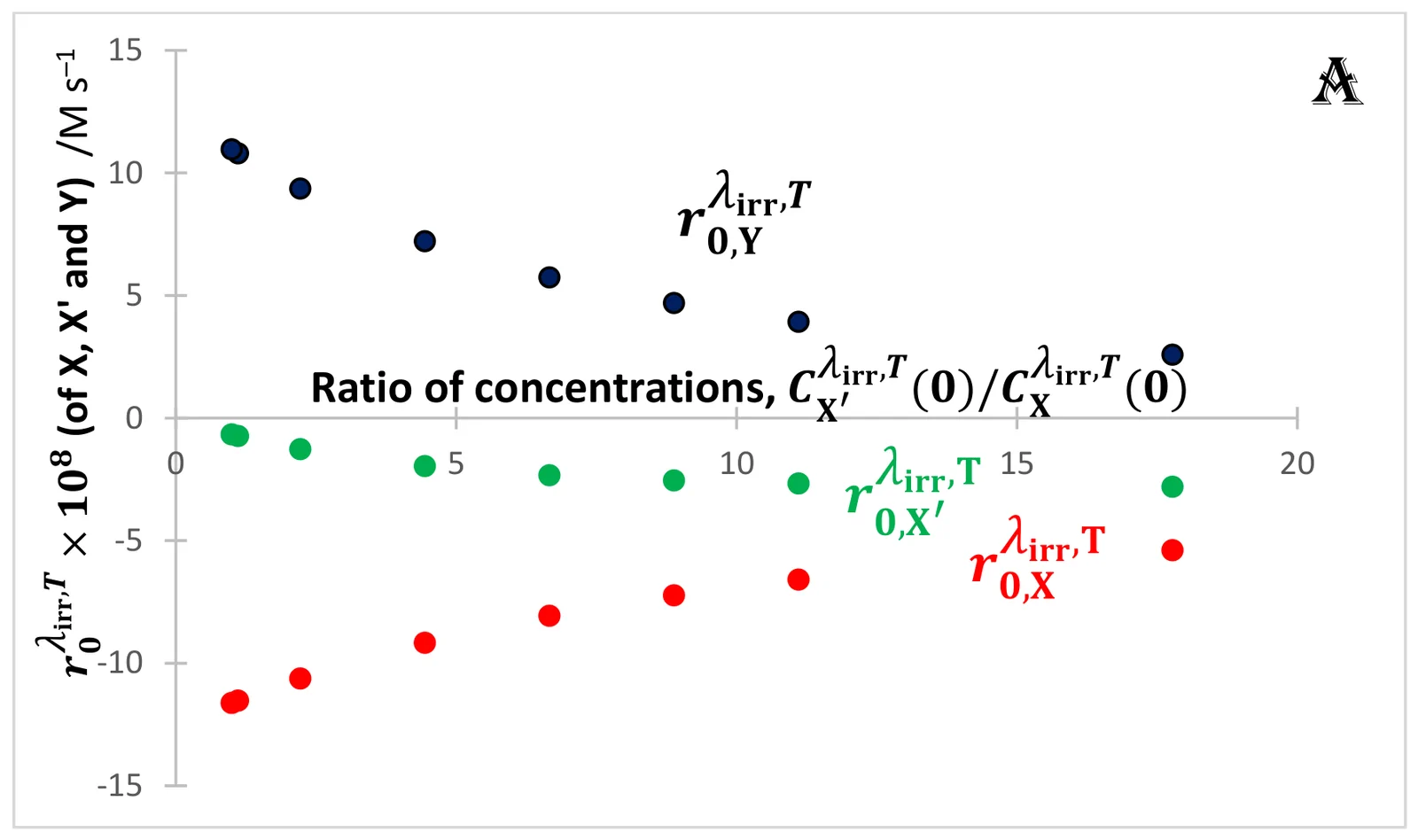

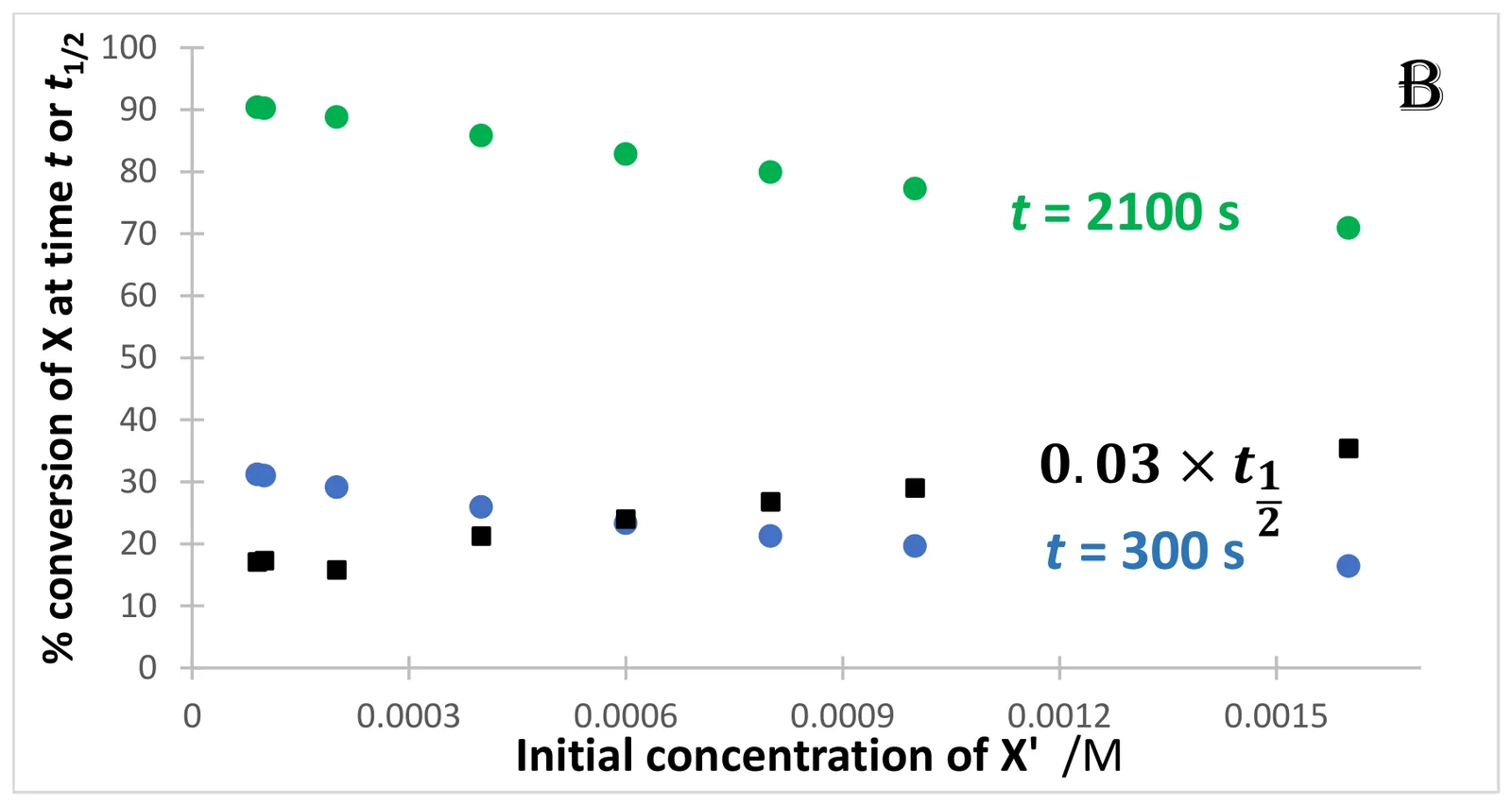
(**A**) Evolution of the initial rates of $X,X^{\prime }\;and\;Y$ with an increase in the second reactant $C_{X^{\prime }}^{\lambda_{irr},T}\left(0\right)$ (under monochromatic irradiation). The same values for extrinsic and intrinsic parameters as for the reaction presented in Figure 2, except that $C_{X^{\prime }}^{\lambda_{irr},T}\left(0\right)$ takes the values $9\times 10^{-5},\;10^{-4},\;2\times 10^{-4},\;4\times 10^{-4},\;6\times 10^{-4},\;8\times 10^{-4},\;10^{-3},\;and\;1.6\times 10^{-3}\;M.$ (**B**) Variation in both the % conversion of reactant X ($\%Conv\left(X\right)=100-100\times C_{X}^{\lambda_{irr},T}\left(t_{s}\right)/C_{X}^{\lambda_{irr},T}\left(0\right)$) at the selected times $t_{s}=300\;and\;2100\;s$ and the half-life time (in s) of the reaction at the indicated values of $C_{X^{\prime }}^{\lambda_{irr},T}\left(0\right)$.

The above photokinetic explanation is translated for the apparent values of the initial rates of the equations. For the example of the reaction $XX^{\prime }YY^{\prime }(\Phi ,k)$, as shown in Figure 5A, when increasing the value of $C_{X^{\prime }}^{\lambda_{irr},T}\left(0\right)$ (while $C_{X}^{\lambda_{irr},T}\left(0\right)$ remains constant), the value of the initial rate of Y ($r_{0,Y}^{\lambda_{irr},T}$) gradually decreases, hence, following the pattern of PKF(0).

Concomitantly, the value of $r_{0,Y^{\prime }}^{\lambda_{irr},T}$ (which is equal to $-r_{0,X^{\prime }}^{\lambda_{irr},T}$) undergoes an increase for increasing values of $C_{X^{\prime }}^{\lambda_{irr},T}\left(0\right)$ (as imposed by the evolution of $A_{X^{\prime }}^{\lambda_{irr},T}\left(0\right)\times PKF(0)$, when $C_{X}^{\lambda_{irr},T}\left(0\right)$ and the other reaction features are unchanged, and $C_{X}^{\lambda_{irr},T}\left(0\right)\leq C_{X^{\prime }}^{\lambda_{irr},T}\left(0\right)$).

The pattern of $r_{0,X}^{\lambda_{irr},T}$ undergoes, for the considered reaction and its features, a reduction in value with an increase in $C_{X^{\prime }}^{\lambda_{irr},T}\left(0\right)$. Here, the ratio $k_{bim}C_{X^{\prime }}^{\Delta \lambda ,T}\left(t\right)/\Phi_{X\to Y}^{\lambda_{irr}}$, for the concentration values considered, varies between 0.06 and 0.7, indicating that the evolution of $r_{0,X}^{\lambda_{irr},T}$ tends to follow the trend imposed by PKF(0) (which decreases with an increase in $C_{X^{\prime }}^{\lambda_{irr},T}\left(0\right)$). This is confirmed by the application of Equation (8) (which is valid for the set of data at hand).

A variation in both $C_{X}^{\lambda_{irr},T}\left(0\right)$ and $C_{X^{\prime }}^{\lambda_{irr},T}\left(0\right)$ would induce a change in $r_{0,X}^{\lambda_{irr},T}$ similar to the variation in $A_{X^{\prime }}^{\lambda_{irr},T}\left(0\right)\times A_{X}^{\lambda_{irr},T}\left(0\right)\times PKF$, which increases for higher values of the initial concentrations.

Quantifying reactivity can also be performed by measuring either t12 or any % conversion of X ($\%Conv\left(X\right))$ after a given reaction time. The recorded values of $\%Conv\left(X\right)$ for our $XX^{\prime }YY^{\prime }(\Phi ,k)$ reaction indicate a monotonic decrease with an increase in $C_{X^{\prime }}^{\lambda_{irr},T}\left(0\right)$, irrespective of the time interval selected (Figure 5B)—a result implying a reactivity reduction, which agrees with the decrease in the initial rate of X (Figure 5A). The explanation of this phenomenon rests on the reduction in the amount of the incident light absorbed by X as this species competes for the available light with the other absorbing species of the medium, and, therefore, the fraction of light absorbed by X lessens as $C_{X^{\prime }}^{\lambda_{irr},T}\left(0\right)$ gets bigger. Similar phenomena were previously described for unimolecular reactions when the concentration of the spectator molecules is increased [1].

The variation in the half-life time values is more subtle, as it presents a dip at smaller values of $C_{X^{\prime }}^{\lambda_{irr},T}\left(0\right)$, followed by a progressive increase at higher values of the initial concentration of $X^{\prime }$. As a property of Φ-order kinetics, t12 values differ among reactions whose values of $C_{X^{\prime }}^{\lambda_{irr},T}\left(0\right)$ are different. A full photokinetic interpretation of the observed t12 plot is not feasible at this stage because the true integrated rate law of X (and, hence, the formula of t12) is not available (the values of t12 were, here, determined from the traces and Equation (9)).

The variation in the optical path length, $l_{irr}$, induces a change in the reaction species’ absorbances ($A_{tot}^{\lambda_{irr},T}\left(t\right)$). In a sense, this is equivalent to changes in the initial concentrations of both reactants, each being multiplied by the same factor. According to Equation (5), only the total absorbance within the expression holding the power number, $\left(1-10^{-A_{tot}^{\lambda_{irr},T}\left(t\right)}\right)$, will be affected (whereas the ratio of the absorbances in Equation (5) remains invariant with a change in $l_{irr}$). An increase in $l_{irr}$ causes higher values of $A_{tot}^{\lambda_{irr},T}\left(t\right)$ and, hence, higher values of the power number expression in Equation (5), which means higher values of the rate and a speed up of the reactivity. The pattern of the reaction’s faster reactivity (and higher values of the initial rate value) is overall different from that induced by the increase in $C_{X^{\prime }}^{\lambda_{irr},T}\left(0\right)$. The reason behind such acceleration relates to a greater amount of light absorbed by the increased number of the molecules of X in the reactor as $l_{irr}$ gets larger while $P_{0}^{\lambda_{irr}}$ and the initial concentrations remain constant. This trend should asymptotically plateau when $l_{irr}$ becomes relatively (very) large.

### 2.11. The Influence of Temperature and Incident Radiation Intensity

**Figure 6:**
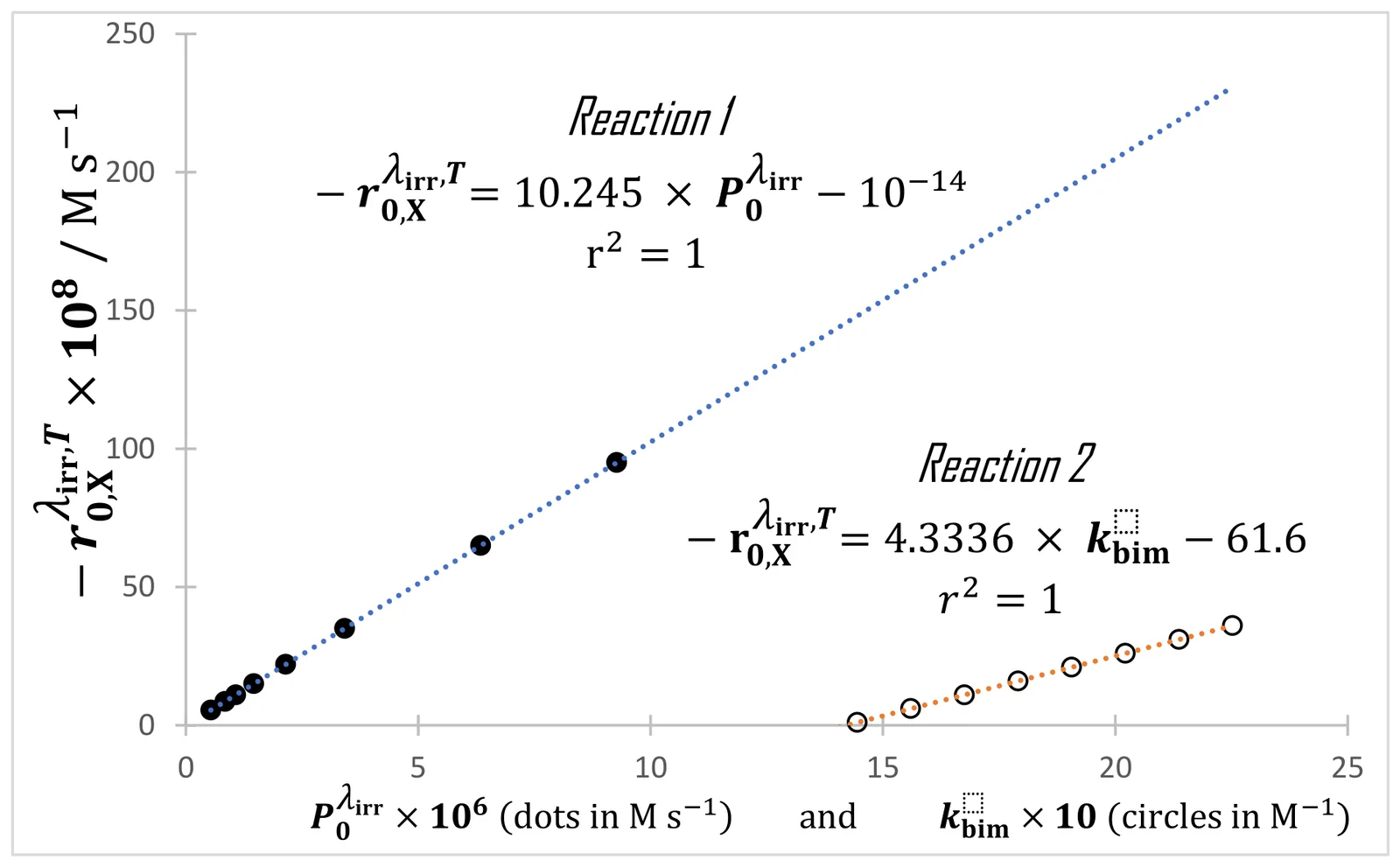
Linear relationships between the initial rate of X and the increasing values of either the incident monochromatic-radiation intensity, $P_{0}^{\lambda_{irr}},$ or the rate constant of the bimolecular photoreaction, $k_{bim}$, for two different $XX^{\prime }YY^{\prime }(\Phi ,k)$ reactions. The reaction features under variable $P_{0}^{\lambda_{irr}}$ values (Reaction 1): $P_{0}^{\lambda_{irr}}=0.5-95\times 10^{-6}\;einstein\;dm^{-3}\;s^{-1},C_{X}^{\lambda_{irr},T}\left(0\right)=10^{-4}\;M,$ $C_{X^{\prime }}^{\lambda_{irr},T}\left(0\right)=3\times 10^{-4}\;M,$ $l_{irr}=0.44\;cm,$ $k_{bim}=0.95\;M^{-1},$ $\Phi_{X\to Y}^{\lambda_{irr}}=2.1\times 10^{-3},$ $\varepsilon_{X}^{\lambda_{irr}}=15,308\;M^{-1}\;cm^{-1},$ $\varepsilon_{X^{\prime }}^{\lambda_{irr}}=7056\;M^{-1}\;cm^{-1},$ $\varepsilon_{Y}^{\lambda_{irr}}=9052\;M^{-1}\;cm^{-1},$ and $\varepsilon_{Y^{\prime }}^{\lambda_{irr}}=13,453\;M^{-1}\;cm^{-1}$; and those for the reaction performed at different medium temperatures (Reaction 2): $k_{bim}=0.1-3.6\;M^{-1},C_{X}^{\lambda_{irr},T}\left(0\right)=10^{-4}\;M,\;C_{X^{\prime }}^{\lambda_{irr},T}\left(0\right)=1.3\times 10^{-4}\;M,P_{0}^{\lambda_{irr}}=3.75\times 10^{-4}\;M^{-1}\;s^{-1},$ $l_{irr}=0.52\;cm,$ $\Phi_{X\to Y}^{\lambda_{irr}}=7.7\times 10^{-4},$ $\varepsilon_{X}^{\lambda_{irr}}=10,334\;M^{-1}\;cm^{-1},$ $\varepsilon_{X^{\prime }}^{\lambda_{irr}}=6753\;M^{-1}\;cm^{-1},$ $\varepsilon_{Y}^{\lambda_{irr}}=11,009\;M^{-1}\;cm^{-1},$ and $\varepsilon_{Y^{\prime }}^{\lambda_{irr}}=16,132\;M^{-1}\;cm^{-1}$.

At higher temperatures, the depletion reaction of $X^{\prime }$ speeds up. Formally, the temperature increase is relayed in Equation (1) by higher values of the rate constant of the bimolecular photoreaction, $k_{bim}$. The overall reaction rate increases with an increase in $k_{bim}$ (Figure 6).

An equivalent proportionality links the absorbed fraction of the radiation by reactant X to the incident radiation intensity ($P_{0}^{\lambda_{irr}}$), and, hence, the rate (Equation (1)) of the photoreactive species increases for higher values of $P_{0}^{\lambda_{irr}}$ (Figure 6). An increase in $P_{0}^{\lambda_{irr}}$ favours the depletion of X and the production of Y.

The composition at the end of the reaction (i.e., the values of $C_{Y}^{\lambda_{irr},T}\left(\infty \right)$ and $C_{Y^{\prime }}^{\lambda_{irr},T}\left(\infty \right)$) varies for a given medium temperature and radiation intensity, but in both cases, the overall reactivity of the bimolecular photoreaction increases with an increase in either or both of these reaction conditions (i.e., T and/or $P_{0}^{\lambda_{irr}}$).

### 2.12. Wavelength Dependent Photoreactivity

As the rate laws of the species involved in bimolecular photoreactions depend on all reaction parameters, both intrinsic and extrinsic, a change in the monochromatic irradiation wavelength entails a change in the absorption coefficients’ values of the species present in the medium and possibly the value of the quantum yield of the direct unimolecular reaction (X→Y). The variation in the latter is well documented for unimolecular and bimolecular photoreactions [1,85]. It is evident that any changes occurring in the absorptivities and/or quantum yield values will automatically affect the photoreactivity and change the overall reaction rate constant and most likely will also alter the composition of the species at the end of the reaction.

The same phenomenon is also expected when one or both of the reactants’ chemical structures are changed (as when studying various species or derivatives). Here, a comparison among the different reactions is difficult and certainly not sufficiently evidenced by an overall parameter such as the half-life time of the reaction.

The advantage of the kinetic elucidation method described in Section 2.9 becomes evident and its use arguably imperative, as it can provide all of the intrinsic parameters of the reaction, which in turn allows for a much more consistent analysis and interpretation of the bimolecular photoreaction behaviour.

### 2.13. Polychromatic Incident Radiation

The predictive modelling of bimolecular photoreactions under polychromatic light irradiation shows analogous behaviours and traces to those observed when the excitation light was monochromatic (see previous sections).

These findings are confirmation that the kinetic traces do not provide relevant information on the nature of the light used (i.e., the type of light used for the reaction cannot be deduced from an analysis of the kinetic traces). The same observation has been stated for unimolecular photothermal reactions [1]. The information about the nature of the light employed needs to be provided by the experimentalist, knowing that a monochromatic beam is only delivered by an irradiation set up that includes a monochromator or when using a laser [1]. If the monochromator is absent from the experimental set up, then the light delivered by either a lamp or an LED is necessarily polychromatic (even if the light emerging from an LED has one colour as visibly apparent to the human eye [86]).

Equations (9), (10) and (12)–(14) are validated for reactions under polychromatic light for the four mechanisms depicted in Scheme 1. The Φ-order character of the kinetics under polychromatic light is, hence, established for bimolecular photoreactions as it was for their irradiation with monochromatic light (see previous sections). It also aligns with a previous statement [78] that can be amended here as: the *Φ*-order kinetic is independent of the nature of the (non-isosbestic monochromatic or polychromatic) light employed for irradiation of unimolecular photo- and photothermal processes both when considered individually or in mixtures, as well as for bimolecular photoreactions. Φ-Order kinetics, hence, stands at the foundation of photokinetics.

The unicity of the equations set adds merit to a harmonious standardisation of the photokinetics of bimolecular photoreactions. As a matter of fact, these equations are the first in the literature to map out the kinetic behaviour of bimolecular photoreactions under either mono- or polychromatic irradiation that have been tested and validated by a rigorous method (Section 2.5) for a large number of reaction conditions and applied to the full extent of the traces.

Different lamps with different profiles will induce different reactivities in a bimolecular photoreaction because the absorptivities of the photoreactive reactant and the other species in the medium will match different sections of the profiles of the incident lights. The effect is even more acute if the quantum yield of X is wavelength dependent. Similarly, it is expected that the reactivity of two bimolecular photoreactions will also be different when subjected to the same lamp (for the same reasons invoked above).

Therefore, without precise knowledge of the intrinsic and extrinsic parameters of the reaction (including the lamp profile), it is difficult to reach reasonable insight into the efficiency of the reaction and/or to achieve its optimisation for the desired application.

Investigation of the reaction under monochromatic light and elucidation of the kinetics become essential tools towards better control of bimolecular photoreactions.

**Figure 7:**
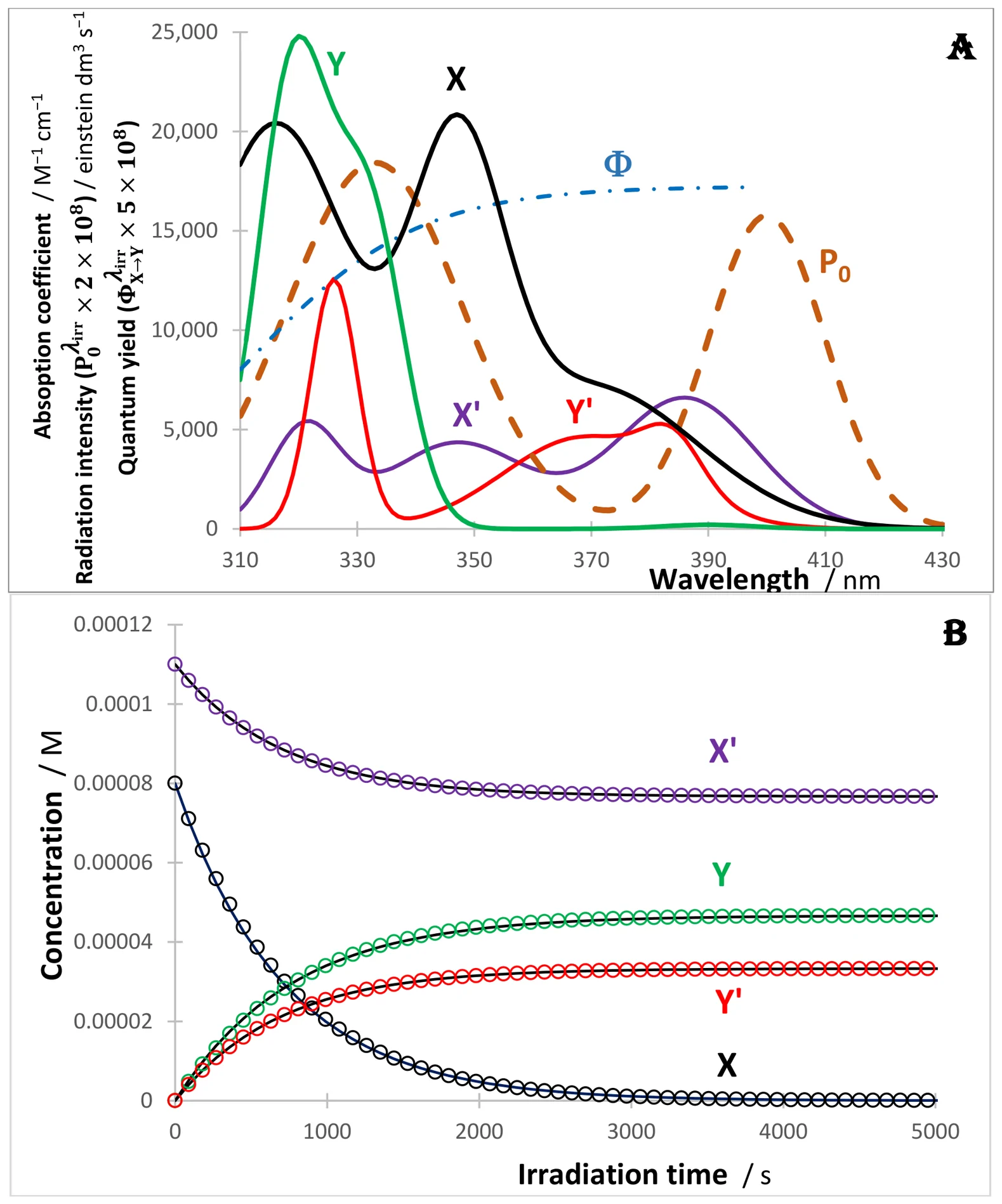
(**A**) Absorption coefficient spectra of the four species of an $XX^{\prime }YY^{\prime }(\Phi ,k)$ bimolecular photoreaction, with $P_{0}^{\lambda_{irr}}$ varying between $5.41\times 10^{-7}$ and $9.22\times 10^{-5}\;eeinstein\;dm^{-3}\;s^{-1},C_{X}^{\lambda_{irr},T}\left(0\right)=8\times 10^{-5}\;M,$ $C_{X^{\prime }}^{\lambda_{irr},T}\left(0\right)=1.10\times 10^{-4}\;M,$ $l_{irr}=0.36\;cm,$ $k_{bim}=0.21\;M^{-1},$ and $1.61\times 10^{-5}\leq \Phi_{X\to Y}^{\lambda_{irr}}\leq 3.43\times 10^{-5}$. The reaction was performed under a polychromatic light (lamp profile given by the brown dashed line) and with a wavelength-dependent quantum yield of the photoreactive X (blue line). (**B**) The traces of the species (RK-calculated (circles)) are fitted with the appropriate Equations (9)–(14) (lines).

For illustration, the $XXYY^{\prime }(\Phi ,k)$ bimolecular photoreaction is subjected to a polychromatic beam of light that is variably absorbed by both the reactants and products present in the medium (Figure 7A). The wavelength-dependence of the quantum yield of reactant X is characterised by an increasing sigmoid-like pattern. The lamp profile consists of several emission bands spreading over the absorption regions of the reaction species. The concentration traces of the species, as well as those of the total absorbance, were fitted by Equations (9)–(14) (and a combination of those as stipulated by Equation (6) for $A_{tot}^{\Delta \lambda ,T}\left(t\right)$). 

## 3. Conclusions

The findings of the present study irrevocably state that Φ-order kinetics is the most suitable to describe the kinetic behaviour of the four bimolecular photoreactions investigated here (Scheme 1). Their rate laws explicitly involve the radiation intensity delivered to the reactor, as well as the amount of light absorbed by each species in the medium at a given reaction time and at every wavelength over the irradiation domain. A corollary to this proof is the inappropriateness of the kinetic model equations developed for thermal reactions for application to such photoprocesses. The above observations align with those previously established for unimolecular photoreactions, which altogether promote Φ-order kinetics as the best, thus far known, foundation for photokinetics.

The true rate laws of bimolecular photoreactions are virtually all unsolvable analytically by conventional mathematical methods due to the presence of the non-linear and time-dependent photokinetic factor ($(1-10^{-A_{tot}^{\lambda_{irr},T}\left(t\right)}/A_{tot}^{\lambda_{irr},T}\left(t\right)$)—a situation that imposes the necessity of developing new strategies capable of determining the kinetic behaviour of these reactions. The strategy used in the present paper has been proven to work as efficiently for bimolecular photoreactions as it was for unimolecular photoreactions and their mixtures.

The explicit (surrogate) equation models (Equations (9)–(14)) play the role of integrated rate laws for the reactions’ species (X, $X^{\prime }$, Y and $Y^{\prime }$) for any experimental conditions or reaction features as long as the concentrations of the reactants and products fall within the limits of the linearity ranges of their respective absorbance calibration graphs. In practice, the latter condition should be satisfied for organic materials when $A_{tot}^{\lambda_{irr},T}\left(t\right)<0.5.$

The validity of the explicit models was proven by testing them on a large set of reaction cases where intrinsic and extrinsic reaction parameters were extensively varied for the studied reaction mechanisms.

The advantages that the explicit model equations bring to the field can be laid out as follows: are a single set applicable to the four reactions depicted in Scheme 1, easy to implement, applicable when irradiation is either performed by monochromatic or polychromatic light, stand in for/play the role of the integrated rate laws, allow for building an efficient kinetic elucidation method, offer the best investigative tools to date, and contribute to the standardisation of photokinetics.

In general, bimolecular photoreactions are slowed down and speeded up, respectively, when the initial concentrations of the reactants, either X or $X^{\prime }$ (with, in the latter case, $k_{bim}C_{X^{\prime }}^{\Delta \lambda ,T}\left(0\right)/\Phi_{X\to Y}^{\lambda_{irr}}\gg 1$), and the optical path length of irradiation ($l_{irr}$) increase or when there is an increase in $C_{X^{\prime }}^{\Delta \lambda ,T}\left(0\right)$, where $k_{bim}C_{X^{\prime }}^{\Delta \lambda ,T}\left(0\right)/\Phi_{X\to Y}^{\lambda_{irr}}\ll 1$. Reactivity is faster at higher temperatures and higher radiation intensities. The changes in reactivity are indicated by the variation in the initial rate of the species that can be obtained from fitting the corresponding concentration trace with the corresponding equation.

Comparison of the performances of the bimolecular photoreactions, either when the experimental conditions are varied or when different reactants are used, can only be objective if it is based on comparing the intrinsic parameters ($k_{bim}$, $\Phi_{X\to Y}^{\lambda_{irr}}$ and $\varepsilon^{\lambda_{irr}}$) of the reactions, i.e., when they are obtained from the kinetic elucidation method.

Therefore, the optimisation of the reactivity of these reactions requires the tuning of their extrinsic parameters by knowing their intrinsic ones.

The findings of the present study are valuable for many fields. They can be exploited in pharmaceutics, photochromes, sunscreens, photocatalysis, and continuous-flow photochemistry, and for predicting yields in solar reactors.

## Figures and Tables

**Table 1:** Application of the elucidation method to solve the kinetics of $XX^{\prime }YY^{\prime }(\Phi ,k)$ driven by monochromatic light.

	Original or Theoretical Data	Equation	Elucidation Using RK Data ^╪^ (% Error)	Elucidation Using Fitting Data (% Error)
Extrinsic parameters *				
$C_{X}^{\lambda_{irr},T}\left(0\right)/M$	$1\times 10^{-4}$	−	−	−
$C_{X^{\prime }}^{\lambda_{irr},T}\left(0\right)/M$	$1.25\times 10^{-4}$	−	−	−
$P_{0}^{\lambda_{irr}}/M^{-1}\;s^{-1}$	$3.75\times 10^{-4}$	−	−	−
$l_{irr}\;/\;\mathrm{cm}$	0.52	−	−	−
Intrinsic parameters				
From the individual X and $X^{\prime }$ species solutions				
$\varepsilon_{X}^{\lambda_{irr}}/M^{-1}\;{cm}^{-1}$	10,334	Calibration graphs	10,334	−
$\varepsilon_{X^{\prime }}^{\lambda_{irr}}/M^{-1}\;{cm}^{-1}$	6753		6753	−
From the unimolecular photoreaction of X under monochromatic light				
$r_{X}^{\lambda_{irr},T}\left(0\right)/M^{-1}\;s^{-1}$	$-2.0497\times 10^{-7}$		$-2.0497\times 10^{-7}$ (−)	$-2.0781\times 10^{-7}$ (1.38)
$\Phi_{X\to Y}^{\lambda_{irr}}$	0.00077	(18)	0.00077 (−)	0.0007714 (0.18)
$\varepsilon_{Y}^{\lambda_{irr}}/M^{-1}\;{cm}^{-1}$	11,009	Calibration graph	11,009	−
From the bimolecular photoreaction under monochromatic light				
$r_{0,X}^{\lambda_{irr},T}/M^{-1}\;s^{-1}$	$-1.44453\times 10^{-7}$	(1)	$-1.44453\times 10^{-7}$ ($8\times 10^{-8}$)	$-1.44496\times 10^{-7}$ (0.03)
$k_{bim}/M^{-1}$	0.1	(19)	0.1 (−)	0.102 (1.86)
$r_{0,Y}^{\lambda_{irr},T}/M^{-1}\;s^{-1}$	$1.42126\times 10^{-7}$	(3)	$1.42399\times 10^{-7}$ (0.18)	$1.42126\times 10^{-7}$ (0.19)
$r_{0,Y^{\prime }}^{\lambda_{irr},T}/M^{-1}\;s^{-1}$	$2.3075\times 10^{-9}$	(4)	$2.3075\;\times 10^{-9}$ (−)	$2.3053\times 10^{-9}$ (0.18)
$r_{{0,X}^{\prime }}^{\lambda_{irr},T}/M^{-1}\;s^{-1}$	$-2.3075\times 10^{-9}$	(4)	$-2.3075\times 10^{-9}$ (−)	$-2.3053\times 10^{-9}$ (0.19)
$C_{Y}^{\lambda_{irr},T}(\infty )/M$	$9.84026\times 10^{-5}$	(23)	$9.84054\times 10^{-5}$ (0.003)	$9.8376\times 10^{-5}$ (0.027)
$C_{Y^{\prime }}^{\lambda_{irr},T}(\infty )/M$	$1.59744\times 10^{-6}$	(24)	$1.59744\times 10^{-6}$ (1.57)	$1.60114\times 10^{-6}$ (0.23)
$C_{X^{\prime }}^{\lambda_{irr},T}(\infty )/M$	$1.2340\times 10^{-4}$	(25)	$1.2342\times 10^{-4}$ (0.021)	$1.23399\times 10^{-4}$ (0.002)
$A_{tot}^{\lambda_{irr},T}(\infty )$	1.01	(6)	1.0101 (0.0016)	1.0099 (0.013)
$\varepsilon_{Y^{\prime }}^{\lambda_{irr}}/M^{-1}\;{cm}^{-1}$	16,132	(28)	16,389.7 (1.59)	16,311.2 (1.11)

^╪^ The elucidation method is applied to the results obtained by RK calculations. * The values of these extrinsic parameters are known.

## Data Availability

The original contributions presented in this study are included in the article. Further inquiries can be directed to the author.
